# Association Between Human Milk-Targeted Metabolites and Maternal Characteristics: Targeted Metabolomic Profiling of Human Milk in Low-Income Settings

**DOI:** 10.3390/metabo16030162

**Published:** 2026-02-28

**Authors:** Sadia Parkar, Nadia Mazhar, Sumera Sharafat, Hamna Ganny, Gul Afshan, Samreen Memon, Khalid Wahab, Aneeta Hotwani, Daniela Hampel, Sidra Kaleem Jafri

**Affiliations:** 1 Department of Paediatrics and Child Health, Aga Khan University, Karachi 74800, Pakistan; sadia.parkar@aku.edu (S.P.); nadia.mazhar@aku.edu (N.M.); sumera.sharafat@aku.edu (S.S.); hamna.ganny2@aku.edu (H.G.); afshan.gul@aku.edu (G.A.); samreen.memon@aku.edu (S.M.); aneeta.hotwani@aku.edu (A.H.); 2 Institute for Global Nutrition, Department of Nutrition, University of California Davis, Davis, CA 95616, USA; dhampel@ucdavis.edu

**Keywords:** human milk composition, targeted metabolomics, lipids, small molecules, maternal characteristics, pathway enrichment

## Abstract

**Background/Objectives**: Human milk (HM) is recognized as the optimal source of infant nutrition, particularly during the first six months of life. While its nutritional aspects and bioactive components are well studied, the HM metabolome remains less understood, particularly in low- and middle-income countries. This study utilized targeted metabolomics for HM profiling and investigated associations of the HM metabolome with maternal and infant characteristics. **Methods**: In total, 267 HM samples and demographic data from mothers participating in the Maternal and environmental Impact assessment on Neurodevelopment in Early childhood years (MINE) study were collected during enrolment (up to 6-months postpartum) and analyzed using the MxP^®^ Quant 500 targeted metabolomics kit from Biocrates. **Results**: A total of 440 metabolites were quantified, mostly lipids such as triglycerides (59.73%), phosphatidylcholines (14.25%), and diglycerides (8.49%), and small molecules including amino acids (26.67%), amino acid-related compounds (21.33%), hexosylceramides (17.33%), and fatty acids (14.67%). Maternal age was positively correlated with a wide range of metabolites, mainly cholesteryl esters, sphingomyelins, triglycerides, and acylcarnitines, while child age was associated with metabolites belonging to acylcarnitine, phosphatidyl-choline, ceramide, diacylglycerol, sphingomyelin, and triglyceride classes. Child’s gender was associated with metabolites, including ceramides, phosphatidylcholines, and sphingomyelins. Pathway enrichment analysis revealed that the metabolites were significantly enriched in valine, leucine, and isoleucine biosynthesis; arginine biosynthesis; phenylalanine, tyrosine, and tryptophan biosynthesis; and glutathione metabolism; however, these reflect annotation-based clustering rather than evidence of active metabolic processes in HM. **Conclusions**: The HM metabolome varies with maternal and infant characteristics, particularly infant age, reflecting cross-sectional differences in milk composition among mother–infant dyads. Enrichment of metabolites annotated to amino acid and antioxidant-related pathways highlights coordinated representation of nutritionally relevant compounds. These findings provide new insight into the factors shaping HM composition in a low- and middle-income populations.

## 1. Introduction

Human milk (HM) is recognized as the optimal source of infant nutrition, particularly during the first six months of life. It plays an essential role not only in providing fundamental macro- and micro-nutrients but also supplies hundreds of bioactive compounds that significantly influence infant health outcomes [1]. These diverse arrays of bioactives compounds have a significant impact on infant growth, immune function, and long-term neurodevelopment. HM is a complex and dynamic matrix, with changing composition across the different stages of lactation. Its intricate matrix and variability renders its analysis challenging and necessitates robust methodologies for the comprehensive identification and quantification of its numerous constituents [2]. The emerging field of HM-targeted metabolomics provides a powerful approach for exploring an array of bioactive compounds, thus offering deeper insights into the metabolic pathways affecting infant growth and development [3].

Targeted metabolomics allows for the simultaneous quantification of hundreds of predefined metabolites, providing a comprehensive analysis of the milk’s biochemical state [4]. This approach offers several advantages, including high sensitivity, reproducibility, and the ability to link specific metabolite concentrations to physiological functions and clinical outcomes. Key classes of metabolites commonly profiled include amino acids, acylcarnitines, biogenic amines, and sphingomyelins, all of which play critical roles in infant metabolism and development [5].

Existing metabolomics research predominantly relies on a compound-based approach, employing various specialized methodologies to analyze individual classes of compounds. For example, ceramides, bioactive sphingomyelins implicated in cell signaling and metabolic regulation, are most often measured using liquid chromatography coupled with tandem mass spectrometry (LC–MS/MS), which provides sensitive and specific detection of individual ceramide species [6]. Other lipid species, including triglycerides, are crucial and environmentally sensitive components of HM, and have traditionally been analyzed by gas chromatography (GC) or nuclear magnetic resonance (NMR) [7]. Amino acids, essential contributors to infant growth and development, have been commonly quantified using high performance liquid chromatography (HPLC) with pre-column derivatization and fluorescence detection, or more comprehensively by LC–MS/MS [8,9,10].

Alternatively, the Biocrates platform offers quantification of various compound classes in only one methodological approach producing comprehensive targeted metabolomics data to further explore the maternal–milk–infant triangular relationships. Maternal characteristics such as dietary intake, body mass index, age, and health status have been shown to significantly influence the HM metabolite profile [11]. Additionally, socioeconomic factors and environmental exposures can contribute to variability in HM composition [12], while maternal obesity and metabolic conditions can affect lipid and amino acid profiles in milk [13]. While the methodologies including HPLC, LC-, or GC-MS are highly informative for their respective targets, they often provide limited understanding of the complex interplay between different metabolic pathways and the holistic composition of HM in low and middle income countries (LMICs) [14]. Although previous studies have described the HM metabolome in high-income populations, little data about the HM metabolome in low- and middle-income countries is available. In addition, there is limited knowledge on how maternal and infant factors impact the HM metabolome in resource-constrained environments. This study addresses these gaps by providing targeted metabolomic profiling of HM from a low-income population and examining associations with key maternal and infant factors.

The objective of the present study was to use comprehensive targeted metabolomics analysis of HM from an LMIC, employing the Biocrates MxP^®^ Quant 500 kit (biocrates life sciences AG, Innsbruck, Austria) to examine associations between HM metabolites and maternal and perinatal characteristics. Specifically, the effects of infant age and sex, as well as maternal factors including age, body mass index, delivery mode, and breastfeeding practices were assessed on the HM metabolome.

## 2. Materials and Methods

### 2.1. Study Design and Setting

This study utilized HM samples collected at enrolment from participants who are part of an ongoing study conducted from July 2022 to July 2026 titled Maternal and environmental Impact assessment on Neurodevelopment in Early childhood years (MINE) (ERC Number: 2022-7181-21273) [15]. The original cohort is a part of a larger longitudinal study aimed at assessing child growth and neurodevelopment, but for the present study, only cross-sectional data from enrolment visits were used.

The original ongoing cohort enrolls mothers and infants and maps trajectories of child growth and neurodevelopment from 1-month to 3.5 years. These participants were recruited from the two peri-urban areas of Karachi. Ibrahim Hyderi and Rehri Goth are the two surveillance sites that have homogeneous low-income communities situated in Bin Qasim, a town in the coastal region of Karachi. Thus, this study is cross-sectional where different mother–infant dyads were enrolled at three infant ages, and a single HM sample was collected at approximately 1-month, 3-months, or 6-months.

### 2.2. Study Participants and Recruitment

The participants were recruited according to the study’s inclusion and exclusion criteria. Mothers who had singleton infants aged 1-month (±30 days), 3-months (±30 days) or 6-months (±30 days) at the time of enrolment were eligible. Dyads were excluded when infants were suspected of having congenital anomalies during clinical visits, a history of hypoxic–ischemic insult, late cry at birth, oxygen support, or hospitalization. Other exclusion criteria were maternal use of antidepressant or antiepileptic drugs in pregnancy, maternal smoking, and fetal abnormalities detected by ultrasound (ventricle enlargement and kidney-forming, gastrointestinal malformations). Further, pregnancy related comorbidities such as pre-eclampsia, hypertension or diabetes mellitus were excluded.

### 2.3. Sample Size

The sample size in the original cohort was calculated based on feasibility; thus, a formal priori calculation of sample size was not done. A total of 270 mother-infant dyads were recruited during enrollment in the parent study. For this secondary analysis, only dyads that provided a HM sample and completed demographic data were included, which resulted in the final sample size of 267 mother–infant dyads.

### 2.4. Participant Recruitment and Study Procedures

A monthly list of households with newborns was obtained from the surveillance (PRISMA) study team. After identifying households with infants of the appropriate age, mother–child dyads were selected using simple random sampling to minimize sampling bias [15]. Research staff visited the mothers at home and administered a screening questionnaire covering both maternal and infant medical histories to determine eligibility according to predefined exclusion criteria.

Following the provision of written informed consent by the parent or legal guardian, the enrolment visit was conducted. A unique study participant identification number (ID) was assigned to each dyad, with both mother and infant sharing the same ID.

Demographic information, maternal physical and mental health assessments, child medical history, physical health evaluations, developmental assessments, and biological samples were collected at the primary healthcare center (PHC) study site. Multiple quality control procedures were implemented to ensure data consistency and reliability throughout the study period. The quality of biological samples was monitored by the laboratory team following standard laboratory protocols. The Data Management Unit (DMU) conducted frequent quality reviews and audits to ensure data integrity across all collected datasets.

### 2.5. HM Samples Collection

HM collection was performed in the PHC by trained research assistants using a standardized protocol to maintain sample quality and minimize contamination. Mothers were instructed not to apply any ointments or topical agents before collection. Milk was manually expressed, guided by trained staff, using gentle massage and a rhythmic press-release technique to encourage milk flow from multiple ducts. Prior to expression, the breast area was cleansed using mild soap and warm water, then dried with a clean, single-use towel. Milk collection began immediately upon manual expression, without discarding initial milk, thereby capturing foremilk and early mid-milk fractions. A minimum of 25 mL of HM was collected directly into sterile, red-top containers, avoiding the use of intermediate vessels and stored at 2–8 °C in pre-cooled Coleman boxes containing fresh ice packs. Three aliquots of 5 mL each were labeled “US” and one 5 mL aliquot was labeled “Spain.” For samples exceeding 20 mL, the same aliquoting was performed, with an additional 5 mL aliquot labeled “Extra Milk.”

### 2.6. Targeted Quantitative Metabolomic Analysis

Metabolomic sample measurement was carried out at biocrates life sciences ag, Innsbruck, Austria, utilizing their MxP^®^ Quant 500 kit (https://biocrates.com/mxp-quant-500-kit/, accessed on 20 December 2025). Most lipid classes and hexoses were measured by flow injection analysis-tandem mass spectrometry (FIA-MS/MS), while small molecules and free fatty acids were analyzed by liquid chromatography-tandem mass spectrometry (LC-MS/MS) using an 5500 QTRAP^®^ mass spectrometer (ABSciex, Darmstadt, Germany), equipped with an electrospray ionization (ESI) source and employing multiple reaction monitoring (MRM) mode for detection. The sample preparation and analysis were carried out according to the manufacturer’s protocol with few adaptations to accommodate the human milk matrix as previously described [16]. Data review and quantification were done using biocrates’ WebIDQ software (cloud-based platform; biocrates life sciences ag). Details of the method, procedure and instrumentation underlying this commercial assay are described in patents EP1897014B1 and EP1875401B1 [17,18]. A full list of metabolites with abbreviations is provided in Supplementary Table S1.

### 2.7. Maternal and Child Characteristics

At enrolment, detailed demographic and socioeconomic information was collected through structured interviews with the mother using standardized questionnaires. These details include child gender, age, maternal and paternal age, education and occupation, and some questions related to breastfeeding practices. For the present study, maternal characteristics including mother age, delivery type (vaginal/C-section), if mother previously breastfed (yes/no), body mass index (BMI), household income (pkr), and mother education (no formal education/any formal education (received any level of education, including primary, secondary, or higher education)). Questionnaire is added in Supplementary Data.

### 2.8. Statistical Analysis

To ensure robust data, the 80% rule was applied: metabolites were excluded from the analysis if more than 20% of the samples revealed concentrations below the limit of detection (LOD) [2,19]. For the remaining metabolites, <LOD values were imputed by replacing them with the minimum observed positive value for that metabolite, increased by 20% (i.e., Minimum + 0.2 × Minimum) [20]. After confirming the non-normal distribution of metabolites using the Shapiro–Wilk test, the non-parametric Kruskal–Wallis test was used to assess statistical differences. Metabolite concentrations across 1-month, 3-months and 6-months were summarized using medians and interquartile ranges (IQR) and subsequently preprocessed using median normalization (log_2_-transformation), and scaled using auto scaling (mean-centers each metabolite and divides by its standard deviation) to eliminate technical variations and reduce skewness for reliable statistical analysis [2]. Bivariate correlation analysis was used to assess the association of individual metabolites with maternal characteristics. For continuous variables, Spearman’s rank correlation coefficient was used, while for binary variables (e.g., child gender or delivery type), point biserial correlation coefficient was applied. To evaluate the independent associations of specific maternal and infant characteristics with HM metabolites, separate multivariable linear regression models were constructed for each predictor of interest. *p*-values were adjusted for false discovery rate (FDR) using the Benjamini–Hochberg procedure, with FDR  <  0.05 considered for significance [19]. Models were adjusted for infant age, maternal age, BMI, delivery mode, and socioeconomic status. Metabolite Set Enrichment Analysis (MSEA) was performed using Kyoto Encyclopedia of Genes and Genomes (KEGG) human metabolic pathways annotations. Enrichment analysis was conducted using the global test algorithm, which employs a generalized linear model to compute a test statistic (Q-statistic) for each metabolite set. To assess the robustness of the findings, a sensitivity analysis was performed using a stricter threshold for values below theLOD. Metabolites were retained only if ≤5% of samples had concentrations below the LOD. The overall analysis was done using R (version 4.3.1) and RStudio (Posit PBC, Boston, MA, USA) and MetaboAnalystR package (version 3.2.0) for enrichment analysis.

## 3. Results

### 3.1. Participants Overview

HM samples were collected from mother–infant cohorts at 1-month (*n* = 105), 3-months (*n* = 107), and 6-months (*n* = 55) of lactation. A total of 48.5% of the infants were female and the average age of the mothers was 25.4 (±5.4) while BMI was 23.21 (±5.09). A total of 67.41% (*n* = 180) had cesarean section while 20.6% (*n* = 55) had vaginal delivery (Table 1).

### 3.2. HM Metabolite Profile

The MxP^®^ Quant 500 kit covers up to 630 metabolites from 26 biochemical classes, including acylcarnitines, alkaloids, amine oxides, amino acid related, and amino acids.

Most metabolites in any given sample were lipids, predominantly triglycerides which accounted for 59.73% of all lipid classes. Phosphatidylcholines (14.25%) and diglycerides (8.49%) were also notably abundant (Table 2). Among the small molecules, amino acids (26.67%) and amino acid-related compounds (21.33%) were the most prominent, followed by fatty acids (14.67%) and hexosylceramides (17.33%, Table 2).

Among these metabolite classes, many concentrations in HM were below LOD (Table 3), e.g., 7129 of 10,680 acylcarnitine measurements (66.75%) were undetectable. Nucleobases and related, hormones and related, and bile acids had the highest number of undetectable metabolites (93.63%, 89.51%, and 77.05%), while other metabolites such as triglycerides (6.40%), amine oxides (2.62%), cresols (1.12%), amino acids (0.59%), and sphingomyelins (0.19%) were usually detectable within the linear range. Carbohydrates and relatedand vitamins and cofactors had no metabolite for any sample with values below the LOD. 

### 3.3. HM Metabolite Composition with Child Age

Given the dynamic changes in HM composition during lactation, variations in the concentrations of all metabolite classes were examined across all collection time points (Table 3). Significant age-related differences (FDR *p*-value < 0.05) were observed in nearly all metabolite classes, including triglycerides, amino acids, and diglycerides. Triglyceride levels increased notably in 3-month-old infants, while amino acid concentrations were higher at one and two months and declined by three months (FDR *p*-value < 0.05). In contrast, acylcarnitines, carbohydrate-related metabolites and fatty acids remained relatively stable across all age groups.

**Figure 1:**
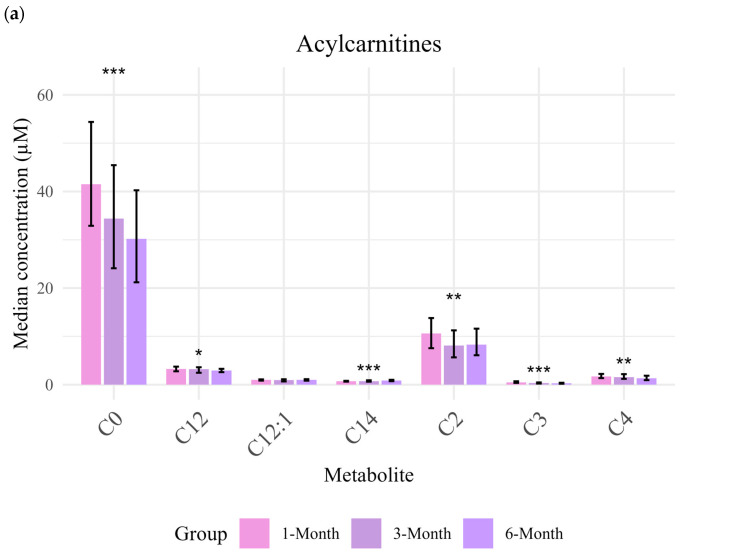

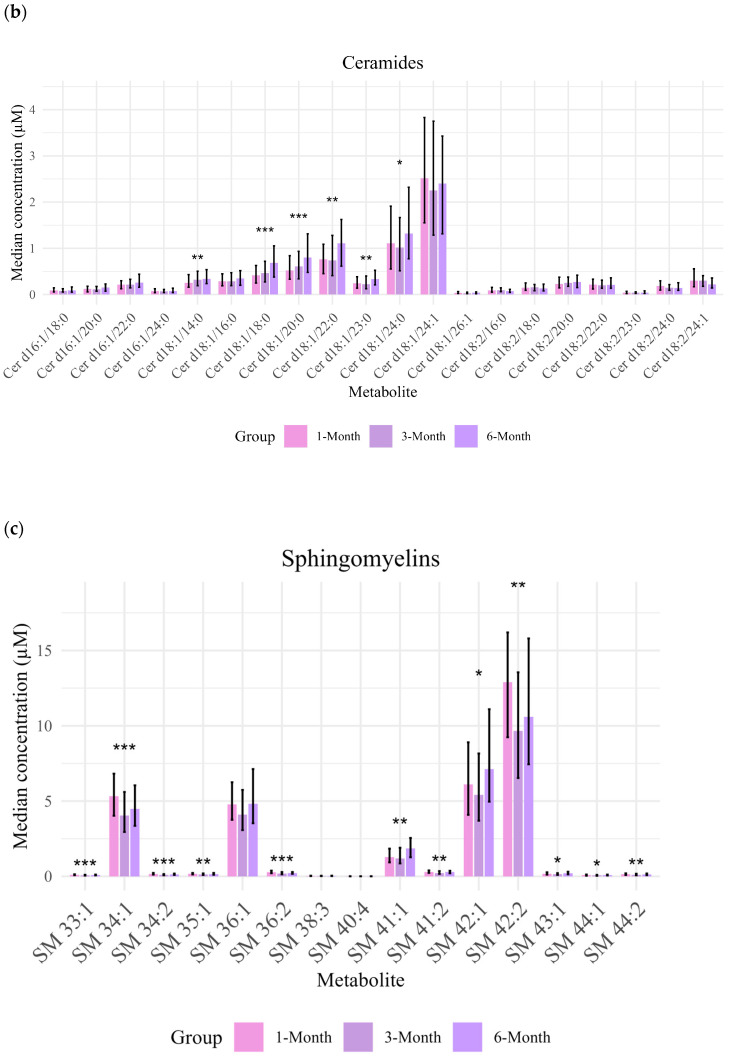

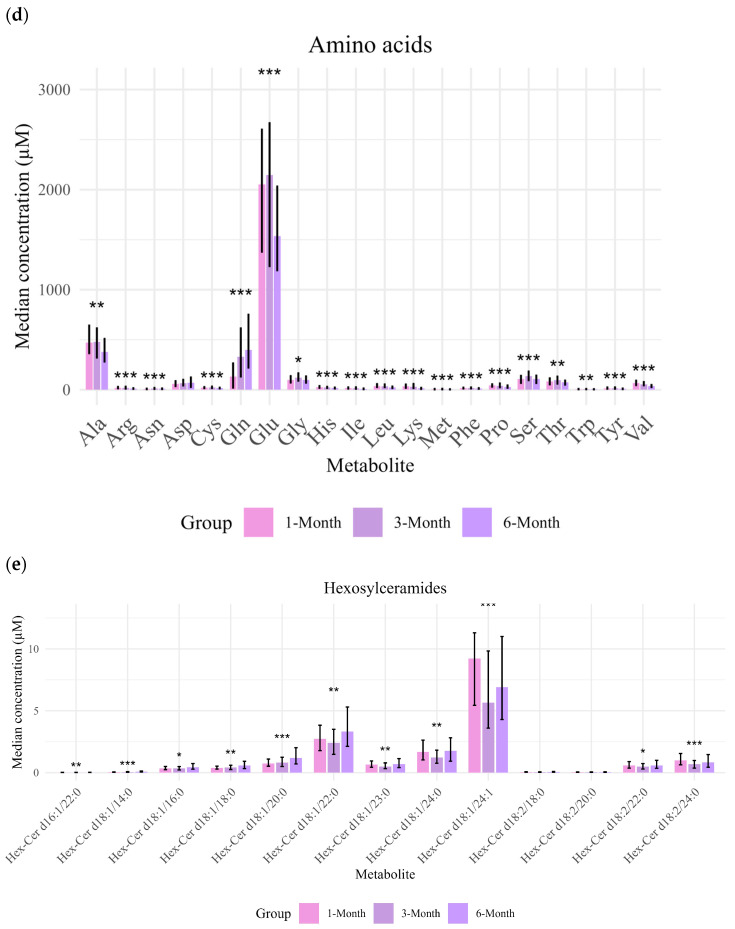

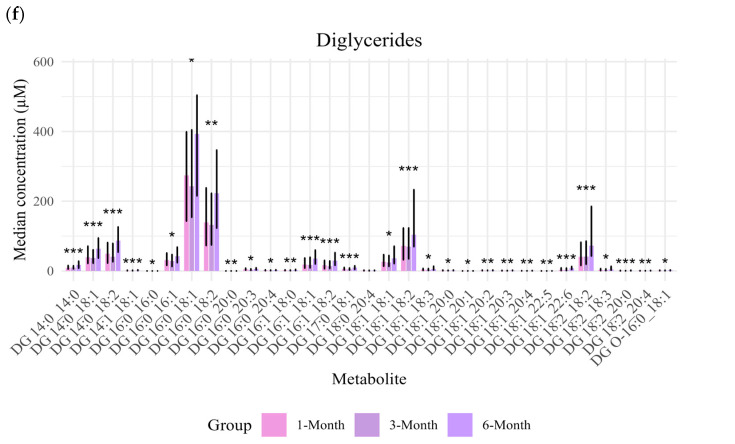
Median concentrations of detectable metabolites (group by class) in milk from Pakistani mothers collected at 1-month (*n* = 105), 3-months (*n* = 106), and 6-months (*n* = 55); (**a**) acylcarnitines, (**b**) ceramides, (**c**) sphingomyelins, (**d**) amino acids, (**e**) hexosylceramides, and (**f**) diglycerides. Significant differences were examined using Kruskal–Wallis test (* *p*-value < 0.05, ** *p*-value < 0.01, *** *p*-value < 0.001).

Variations in metabolites within class were observed for acylcarnitine with declining concentrations over time (Figure 1); particularly, C0, C2, C3, C4, C12, and C14 concentrations were significantly lower at 6-months compared to at 1-month and 3-months (FDR < 0.05) (Figure 1a). Similarly, sphingomyelins (e.g., SM 33:1, SM 34:1, SM 34:2, and SM 36:1) showed lower concentrations at 6-months (Figure 1c). Similarly, ceramides (e.g., Cer d18:1/18:0, Cer d16:1/18:0, and Cer d18:1/20:0) were present in significantly higher amounts at 6-months (Figure 1b).

Significant differences were found in almost all triglycerides (e.g., TG 16:0-32:2, TG 16:0-32:0, TG 16:0-32:1, TG 18:1-30:2, TG 16:0-32:2, and TG 18:0-32:1) with the highest median concentrations of each metabolite at 6-months compared to 1-month (FDR < 0.05) (Figure S1). Similarly, median concentrations of phosphatidyl-cholines such as PC(28:1), PC(30:0), PC(36:1), and PC(36:2) were higher at 6- compared to 3-months (FDR < 0.05) (Figure S2). Amino acids (e.g., Glu, Ala, and His), diglycerides (e.g., DG O-14:0-18:2 and DG 18:1-18:2) (Figure 1d), and hexosylceramides (i.g., Hex-Cer d18:1/18:0, Hex-Cer d18:1/20:0, and Hex-Cer d18:1/22:0) (Figure 1e) revealed significantly higher concentrations at later stages of lactation.

### 3.4. Association of Maternal and Child Characteristics with HM Metabolome Profile

**Figure 2:**
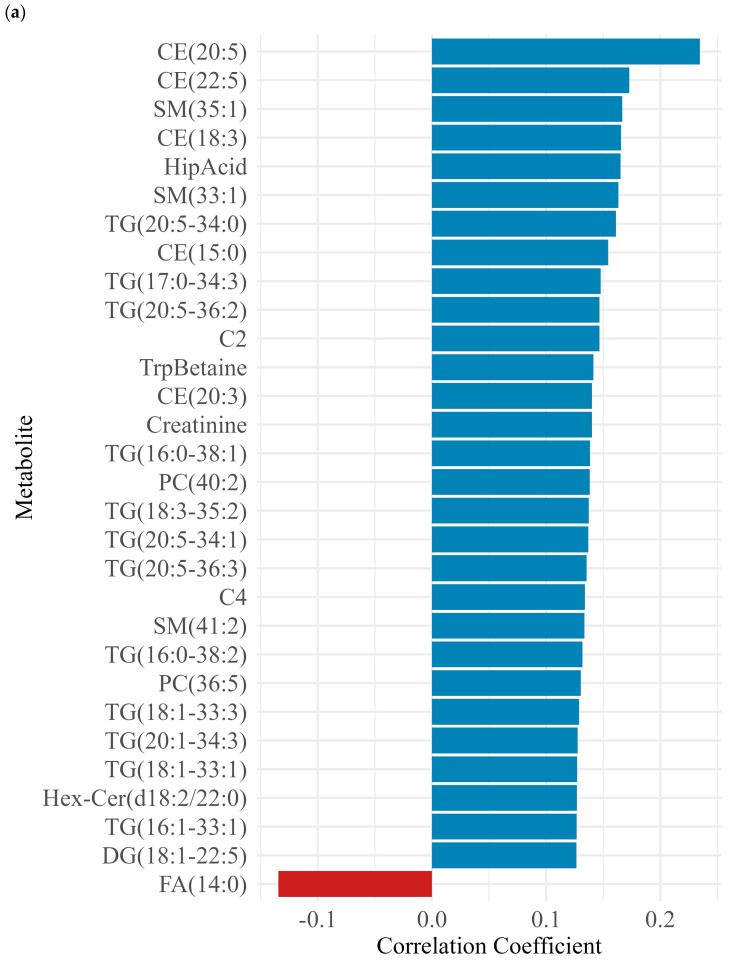

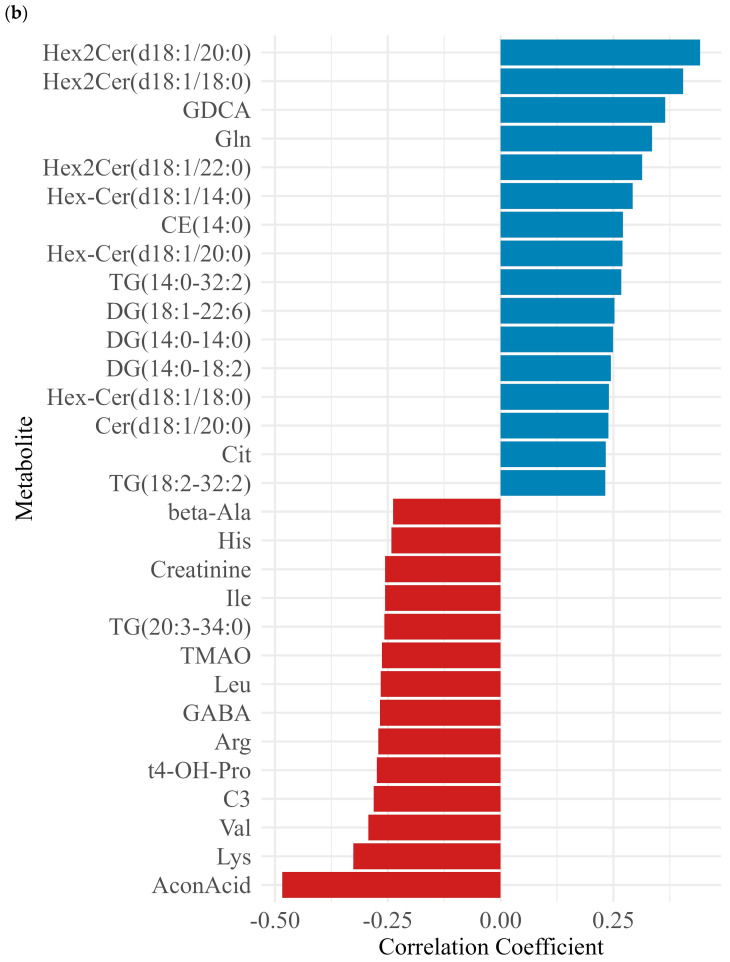

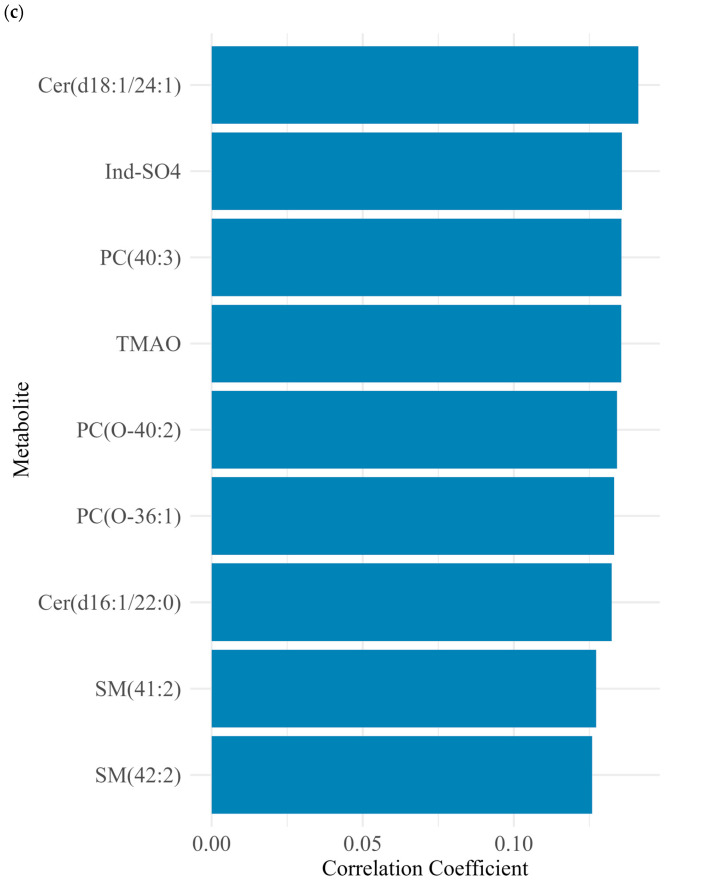

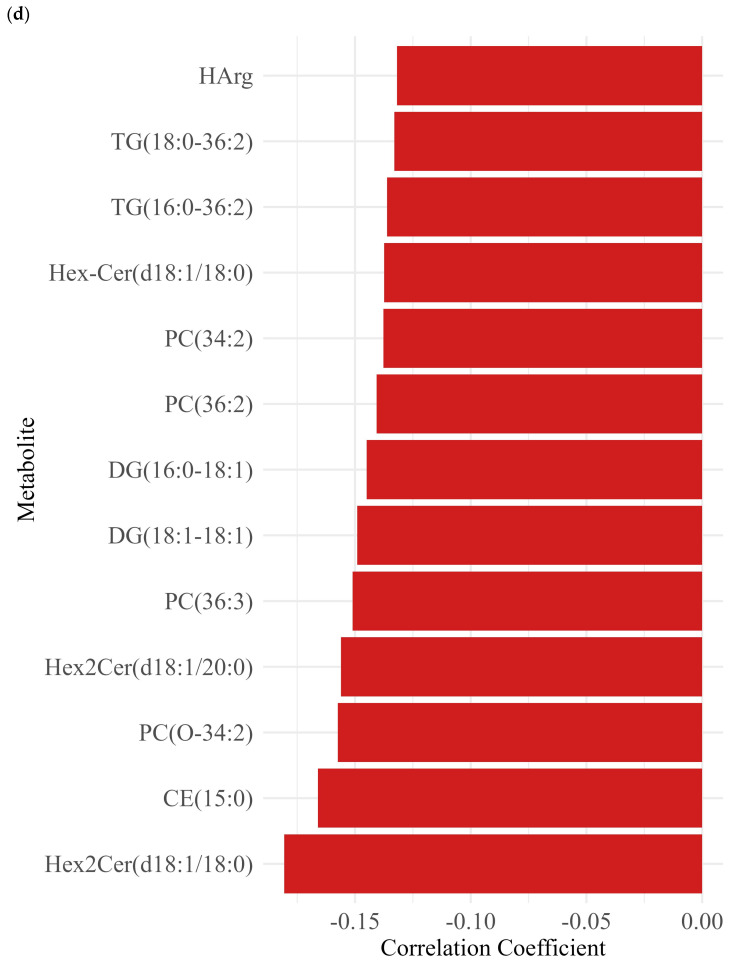

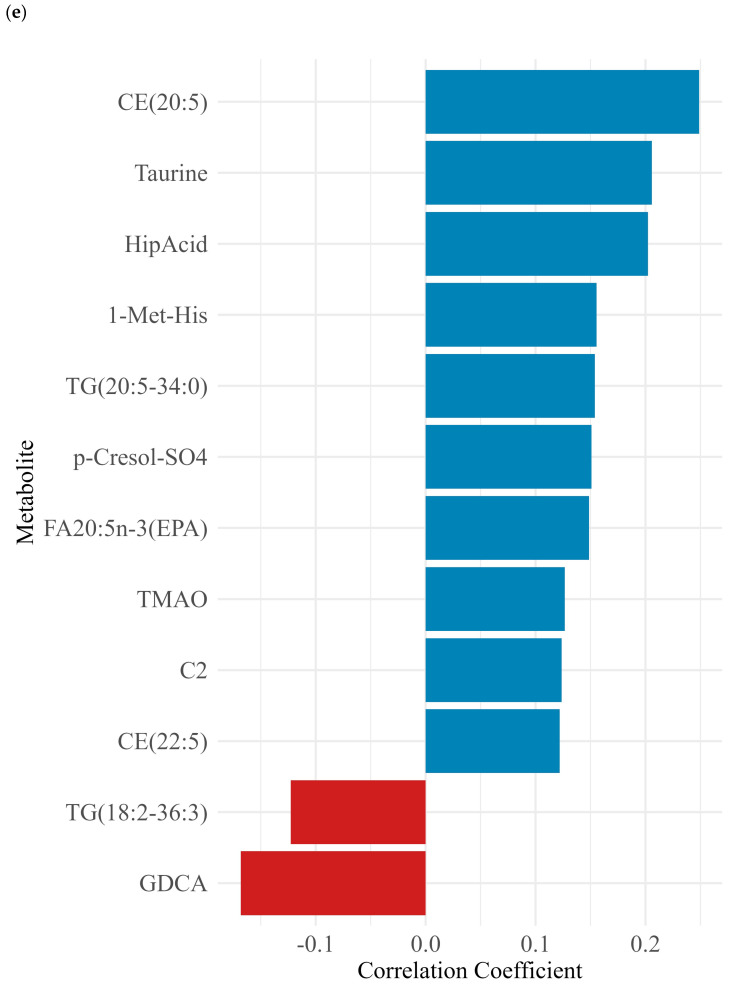

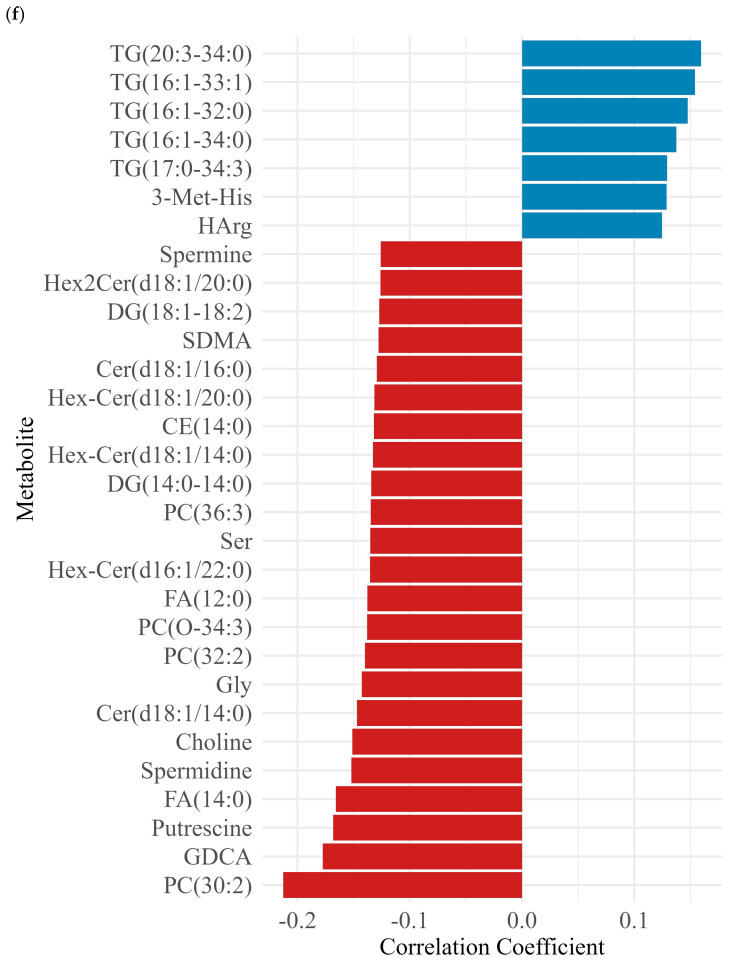

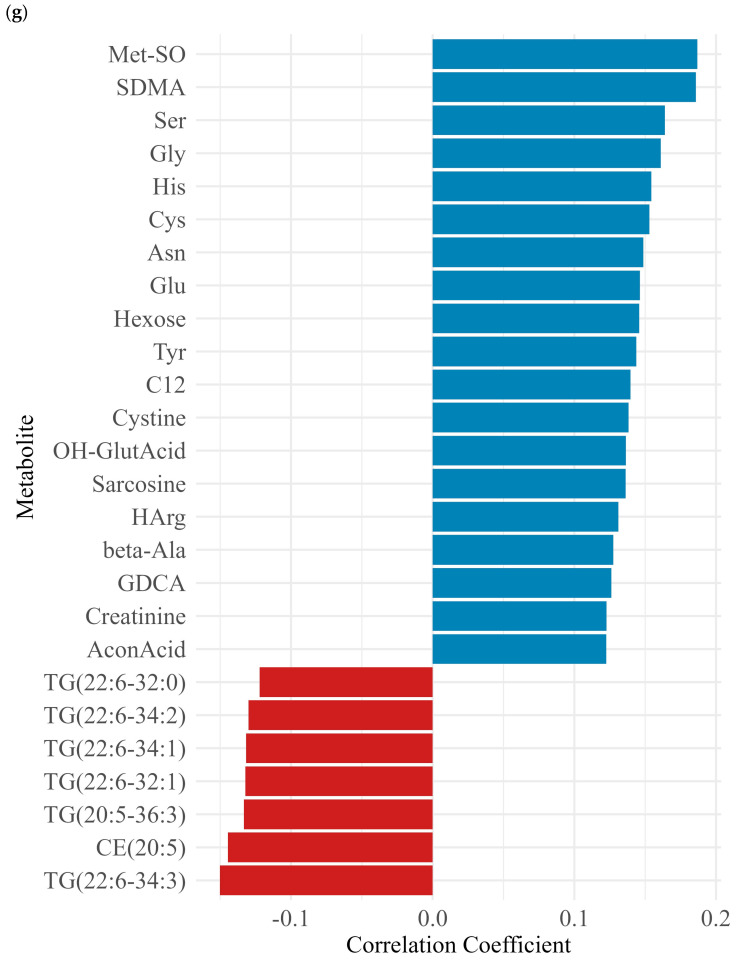

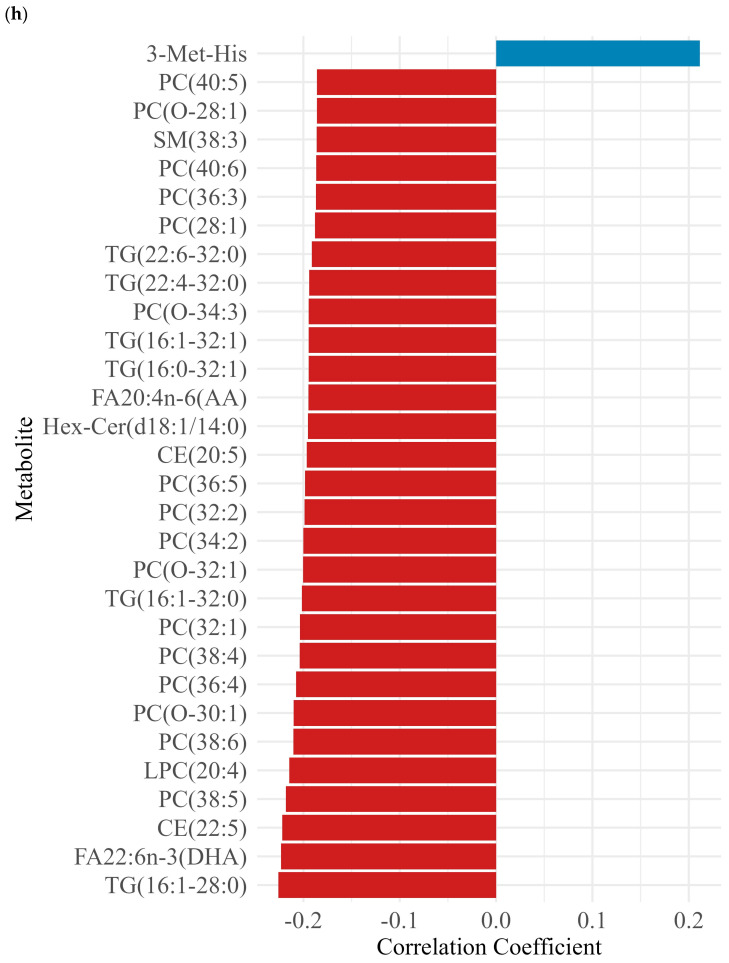
Associations between HM metabolites and maternal and infant characteristics. Metabolites associated with (**a**) age of mother (top 30 metabolites by correlation coefficient), (**b**) age of child (top 30 metabolites by correlation coefficient), (**c**) gender (male (blue) vs. female (red)), (**d**) delivery type (vaginal (blue) vs. C-section (red)), (**e**) previous breastfeeding (no vs. yes), (**f**) maternal BMI (top 30 metabolites by correlation coefficient), (**g**) household income, and (**h**) maternal education (formal education/no formal education) (top 30 metabolites by correlation coefficient). Blue bars are showing positive association and red bars are showing negative association.

Examining the association of maternal characteristics such as mother and child age, gender, delivery type, and number of children mother breastfed (Figure 2) revealed a potential association between metabolites and maternal characteristics. Maternal age showed a significantly positive but weak association with several metabolites, particularly cholesteryl esters (e.g., CE (15:0) (r = 0.15, FDR = 0.04), CE (18:3) (r = 0.17, FDR = 0.04), CE (20:3) (β = 0.14, FDR = 0.04)) and sphingomyelins (e.g., SM(33:1) (r = 0.16, FDR = 0.04), SM(34:2) (r = 0.12, FDR = 0.04), SM(35:1) (r = 0.17, FDR = 0.04) and SM(36:2) (r = 0.12, FDR = 0.04). Triglycerides TG (20:1-32:1), TG (20:1-34:3) and acylcarnitines C2 and C4 also demonstrated positive correlations (Figure 2a). Child age was negatively associated with small molecules, including gamma-amino-butyric acid (GABA) (r = −0.27, FDR < 0.01), aconitic acid (r = −0.48, FDR < 0.01), lysine (r = −0.33, FDR < 0.01) and valine (r =−0.29, FDR = 0).

Selected biogenic amines (beta-Ala and GABA), hexosylceramides (Hex-Cer(d18:1/24:1), and Hex-Cer(d18:2/24:0), acylcarnitines (C2, C3, and C4), phosphatidyl-cholines (PC(O-34:0), PC(O-38:2), and PC(O-38:3)), sphingomyelins (SM(33:1), SM(34:1), SM(34:2), SM(35:1), and SM(36:2)), triglycerides (TG(20:3-34:0)), and amino acids (creatinine, taurine, sarcosine, and taurine) revealed a significant negative correlation with child age (FDR < 0.01). Conversely, positive correlations were observed with ceramides (e.g., Cer(d18:1/20:0), r = 0.24, FDR < 0.01), cholesteryl esters (e.g., CE (14:0), r = 0.27, FDR < 0.01), and diacylglycerols (e.g., DG (14:0-14:0), r = 0.25, FDR < 0.01) (Figure 2b).

Child’s gender was associated with modest differences in selected metabolites including ceramides [Cer(d16:1/22:0), Cer(d18:1/24:1)], phosphatidylcholines [PC(40:3), PC(O-36:1), PC(O-40:2)], sphingomyelins [SM(41:2), SM(42:2)], indoxyl sulfate (Ind-SO_4_), and trimethylamine N-oxide (TMAO), (Figure 2c). Delivery type was negatively associated with several lipid species across multiple classes, including cholesterol ester [CE(15:0)], diacylglycerols [DG(16:0-18:1), DG(18:1-18:1)], dihexosyl-ceramides [Hex2Cer(d18:1/18:0), Hex2Cer(d18:1/20:0), and Hex-Cer(d18:1/18:0)], phosphatidylcholines [PC(34:2), PC(36:2), PC(36:3), PC(O-34:2)], triacylglycerols [TG(16:0-36:2), TG(18:0-36:2)], and homoarginine (HArg) (Figure 2d).

Positive correlations were observed for hippuric acid, taurine, cholesterol esters (CE (20:5), CE (22:5)), EPA (FA20:5n-3), TMAO, p-cresol sulfate, 1-methylhistidine, and triacylglycerols (TG (20:5-34:0)). In contrast, the concentration of glycodeoxycholic acid and triacylglycerols TG (18:2-36:3) were low among mothers who had previously breastfeed (Figure 2e).

Higher maternal BMI was associated with lower levels of several amino acids (e.g., Asn, Gly, and Ser), polyamines (putrescine, spermidine, and spermine), choline, and various lipid species (ceramides, hexosylceramides, diacylglycerols, and phosphatidylcholines) and with higher levels of some triglycerides, including TG(16:1-33:1) (r = 0.154, FDR = 0.049) and TG(20:3-34:0) (Figure 2f). Several HM metabolites showed statistically significant associations with household income; higher household income was positively correlated with higher levels of several amino acids (e.g., asparagine, cysteine, glutamate, glycine, histidine, serine, and tyrosine), small molecules (e.g., beta-alanine, hexose, aconitic acid, hydroxyglutaric acid, sarcosine, creatinine, cystine, HArg, Met-SO, SDMA, and GDCA), and certain lipid species (e.g., C12). Conversely, some triglycerides and cholesteryl ester [CE(20:5)] showed inverse associations with income (e.g., TG(20:5_36:3), TG(22:6-32:0), TG(22:6-32:1), TG(22:6-34:1), TG(22:6-34:2), and TG(22:6-34:3)) (Figure 2g). Maternal education was associated with lower levels of multiple lipid species including some ceramides, cholesteryl esters, phosphatidylcholines, sphingomyelins, and triglycerides (Figure 2h).

In multivariable models adjusted for potential confounders, child age remained significantly associated with a broad range of HM metabolites after FDR correction (FDR < 0.05). Associations were observed across multiple metabolite classes, including triglycerides, phosphatidylcholines, sphingomyelins, ceramides/hexosylceramides, diglycerides, and amino acids. Several lipid species showed positive associations with child age (e.g., Hex2Cer(d18:1/18:0) and Hex2Cer(d18:1/20:0)), while multiple small molecules and amino acids demonstrated inverse associations (e.g., AconAcid, GABA, and branched-chain amino acids such as Val, Leu, and Ile). Overall, effect sizes were modest but consistent in direction across metabolite classes. Meanwhile, for maternal education, adjusted associations were most prominent among lipid classes, particularly phosphatidylcholines and triglycerides. Most phosphatidylcholine species showed inverse associations with maternal education (e.g., PC(38:6), PC(40:6), and ether-linked PC species), whereas triglycerides demonstrated both positive and negative associations depending on fatty-acyl composition. Also, prior breastfeeding experience was significantly associated with triglyceride (e.g., TG(17:2-36:4), TG(18:1-36:3), and TG(20:4-36:3)). In contrast, other variables such as child’s gender and maternal age, BMI, delivery type, and household income showed no significant effect after FDR correction (Supplementary Table S3A–H).

### 3.5. Pathway Enrichment Analysis

**Figure 3:**
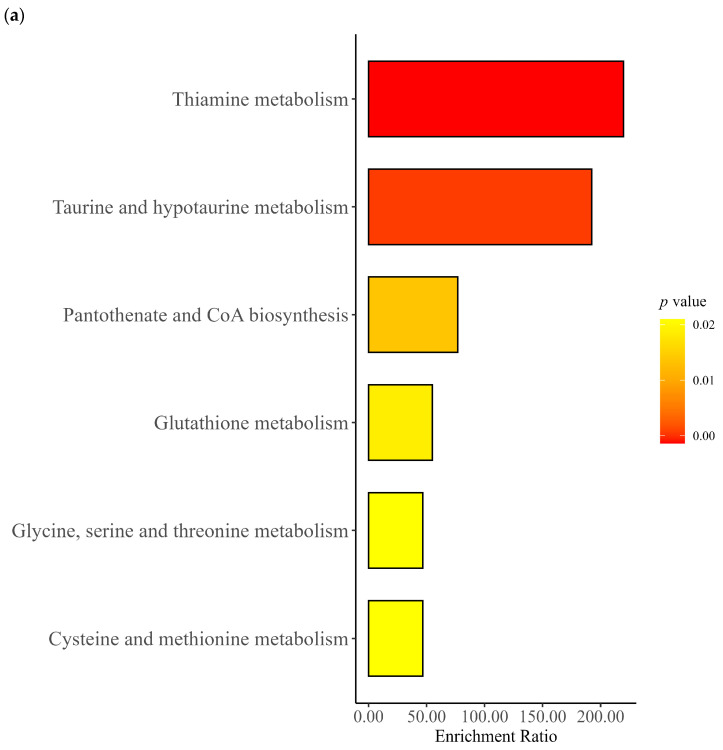

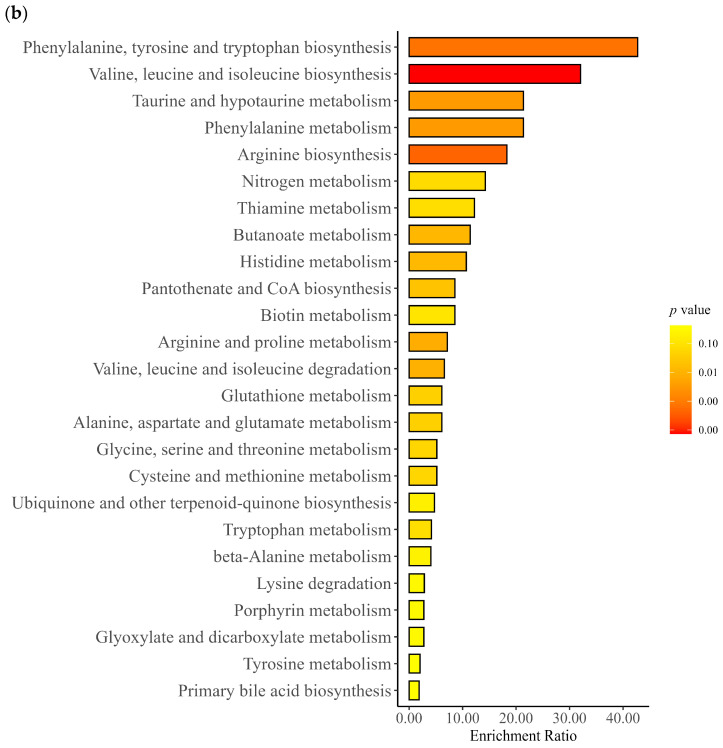

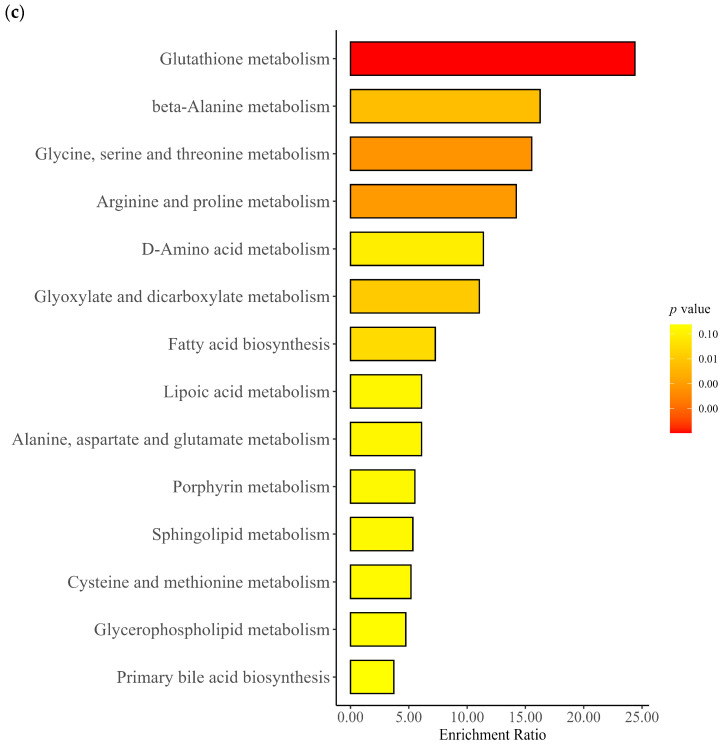

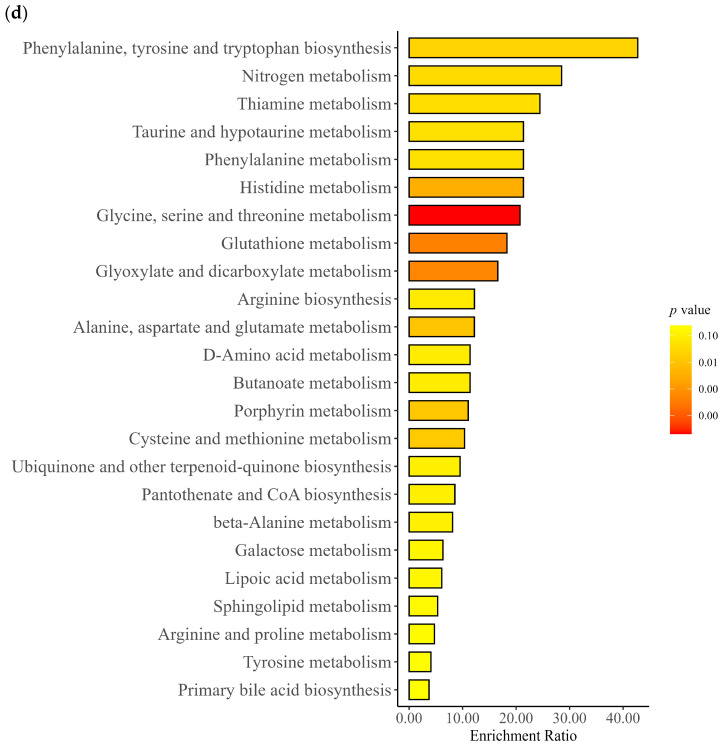

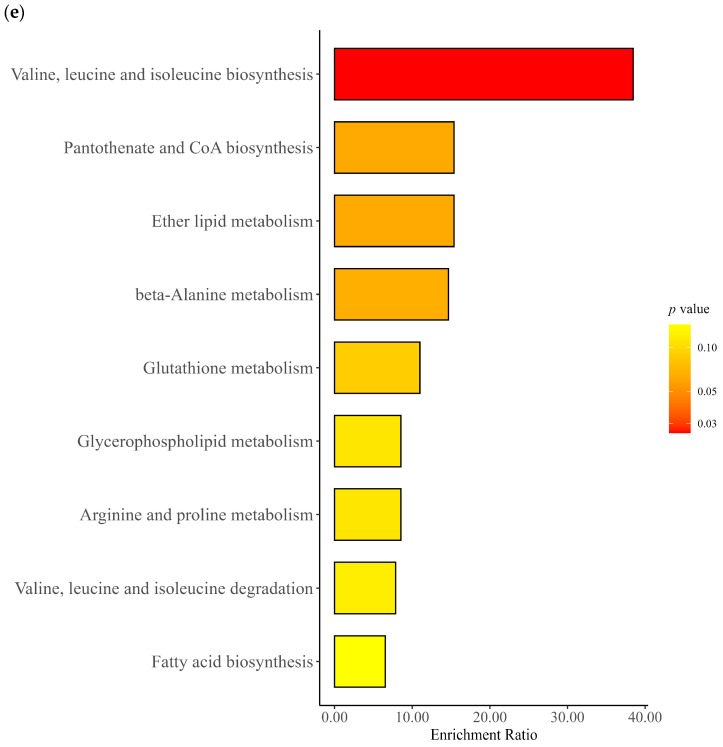
Pathway enrichment analysis of metabolites significantly associated with mother’s perinatal and postnatal factors. (**a**) Maternal age, (**b**) child age, (**c**) maternal BMI, (**d**) monthly household income, and (**e**) maternal education.

Among metabolites significantly associated with maternal characteristics (FDR  < 0.05), pathway enrichment analyses were conducted using KEGG human metabolic pathway annotations. Metabolites significantly associated with mother’s age revealed enrichment of metabolites belonging to valine, leucine and isoleucine biosynthesis; butanoate metabolism; alanine, aspartate and glutamate metabolism; arginine and proline metabolism; valine, leucine, and isoleucine degradation; and fatty acid biosynthesis, but these pathways were not statistically significant after FDR correction (Figure 3a). In contrast, valine, leucine, and isoleucine biosynthesis (FDR = 0.01), arginine biosynthesis (FDR = 0.02), and phenylalanine, tyrosine, and tryptophan biosynthesis (FDR = 0.02) were significantly enriched among metabolites associated with child age (Figure 3b). While other pathways, such as taurine and hypotaurine metabolism, arginine and proline metabolism, valine, leucine and isoleucine degradation, and butanoate metabolism were identified, they were not significantly enriched. For the mother BMI, several pathways showed significant enrichment. Glutathione metabolism was the most significantly enriched (FDR < 0.01), followed by glycine, serine and threonine metabolism (FDR = 0.024) and arginine and proline metabolism (FDR = 0.024) pathways (Figure 3c).

For the metabolites significantly associated with household income, pathway analysis revealed overrepresentation of metabolites belonging to glycine, serine and threonine metabolism (FDR < 0.01), glutathione metabolism (FDR = 0.02), and glyoxylate and dicarboxylate metabolism (FDR  = 0.02) (Figure 3d). Other pathways include histidine metabolism, alanine, aspartate and glutamate metabolism, porphyrin metabolism, and cysteine and methionine metabolism; however, these pathways were not significantly enriched (FDR > 0.05). Among metabolites significantly associated with mothers’ education, pathways such as valine, leucine, and isoleucine biosynthesis, arginine biosynthesis, glycerophospholipid metabolism, nicotinate and nicotinamide metabolism were enriched; however, these results were not statistically significant after FDR correction (FDR > 0.05) (Figure 3e).

### 3.6. Sensitive Analysis

Applying a stricter exclusion criterion (≤5% <LOD) reduced the number of metabolites retained for analysis to 393. However, the overall patterns of association between metabolites and variables including child’s age and gender, mother’s age, BMI, delivery type, breastfeeding, education and household incomes, remained largely consistent with the primary analysis. Specifically, the direction and relative strength of the associations and the classes of metabolites involved were similar in both analyses. The outcomes of sensitive analysis are provided in Supplementary Table S4A–H.

## 4. Discussion

The objective of the study was to assess (a) the targeted metabolomics analysis of HM in a low-income setting and (b) the associations of the HM metabolome with maternal and child characteristics in such settings by employing the Biocrates MxP^®^ Quant 500 kit. By collectively analyzing these components, our study highlights distinct metabolomic patterns in HM across a broad spectrum of constituents.

### 4.1. Human Milk Metabolic Profile

Most metabolites present in HM were lipids, predominantly triglycerides (59.73%), phosphatidylcholines (14.25%), and diglycerides (8.49%). Among small molecules, the most prominent classes were amino acids (26.67%), amino acid-related compounds (21.33%), hexosylceramides (17.33%), and fatty acids (14.67%). These findings are consistent with a study conducted among Spanish mothers which also reported triglycerides to be the predominant lipid class (58%), followed by amino acids (35%) and amino acid-related compounds (32%). However, the fatty acids were found to be much lower (2%) [2].

Variations in metabolites were observed across different metabolite classes, suggesting substantial inter-individual variability in metabolite concentration pertaining to biological factors. Selvalatchmanan et al. also reported moderate-to-substantial inter-individual variation and low variation in sphingomyelins [21]. In milk samples from Danish mothers, lipid-related classes accounted for the majority of variability in HM composition [22]. Similarly, Yuhas et al. reported high variation in fatty acid composition across nine countries, primarily due to differences in DHA levels [23]. This variability may be influenced by factors such as time since last feed, lactation stage, maternal physiology, genetic and hormonal differences, and diet [21,24,25].

### 4.2. Variation in Human Milk Metabolites at Different Lactation Stages

Acylcarnitine, sphingomyelin, fatty acids, carboxylic acid, ceramides, and cholesteryl esters showed significant age-related changes. Among acylcarnitines, C0 (free carnitine) had the highest concentration across all lactation stages (1-, 3-, and 6-months). Notably, C0, C2, C3, and C4 significantly decreased by 6-months compared to earlier time points (1- and 3-months), suggesting a shift in milk composition during lactation, likely reflecting evolving infant needs and maternal physiological adaptations [26]. Such a progressive decline in acylcarnitine levels over 25 weeks of lactation has been previously observed [5]; a notable discrepancy in the dynamics of C0 was observed.

HM from mothers of 6-month-old infants had significantly higher ceramide d18:1/18:0 and d18:1/20:0 levels than those of 1-month-olds. This parallels Albi et al. reporting increased 18:0 Cer and 18:1 Cer (3–12 months) [27], while 24:0 Cer and 24:1 Cer declined from 3-months to 1-year, unlike our observed increasing concentrations in the 6-month group compared to the 1-month group [27].

Our findings of declining sphingomyelins levels during lactation aligns well with previous findings [27] and correlates with alterations in the mammary glands’ sphingomyelins production [28,29]. Fatty acids such as FA 12:0, FA 14:0, FA 18:1, and FA 18:2 showed relatively higher concentrations in milk collected at 6-months compared to 1-month, though the differences were not significant. These evolving changes in HM composition throughout lactation further emphasize the changes in nutritional requirements of infants at different lactation stages [29]. On the other hand, a study involving milk from Malaysian mothers (transitional, early, and mature) identified oleic acid (33%) and palmitic acid (26%) as primary fatty acids. These, plus linoleic (10%) and alpha-linolenic (0.4%) acids, remained stable across lactation stages. These conserved concentrations indicate a prolonged consistent need of these lipids as vital macronutrients and precursors for crucial early infant development and brain maturation [30].

### 4.3. Association of Maternal and Child Characteristics with Milk Metabolome

About 40 metabolites, including ceramides, cholesteryl esters, and sphingomyelins, were positively associated with maternal and child age, indicating age-related shifts in milk lipid composition. While maternal age influenced fatty acid profiles, levels of FA 12:0, FA 14:0, and FA 18:2 were primarily diet dependent. Interestingly, these fatty acid levels were found to be lower in mothers who had cesarean deliveries [31].

While positive associations between maternal age and several lipid-related metabolites were found, a single fatty acid, FA 14:0, was negatively correlated with age. These findings align with previous studies reporting higher lipid concentrations in the early milk of older mothers [32], and support evidence that age-related changes in lipid metabolism during lactation may be influenced by hormonal shifts, breastfeeding frequency, and duration [33]. However, the existing literature lacks empirical evidence regarding the association between maternal age and HM metabolites.

Child’s age was associated with several lipid-related metabolites and small molecules. Untargeted metabolomic studies have reported considerable changes in lipid metabolites and amino acid metabolites with progression of lactation stage, which represent changes in de novo synthesis and nutritional demands of the infant [5]. Lipid-related metabolites such as cholesteryl ester, diacylglycerides, or dihexosylceramides were significantly lower in milk from mothers who delivered via cesarean section compared to vaginal birth. Samuel et al. found that HM from mothers who had vaginal births contained higher levels of various fatty acids as well as higher concentrations of monounsaturated fatty acids (MUFA), calcium, and phosphorus [33]. Our study showed no significant association between delivery type and these metabolites. The difference in findings may be influenced by hormonal, autocrine, and paracrine changes triggered by the mode of childbirth [34].

Lipids such as ceramides (d16:1/22:0, d18:1/24:1), phosphatidylcholines (40:3, O-36:1, O-40:2), and sphingomyelins (41:2, 42:2) were significantly elevated in HM from mothers of male infants. Khelouf et al. reported higher fat content in mature milk from mothers of male infants, while carbohydrate and glucose concentrations were higher for females [35]. However, findings are mixed: Hosseini et al. observed greater total fat content in milk from mothers of female infants, measured by a Lacto-Scan analyzer [36], whereas Bzikowska-Jura et al. found higher carbohydrate levels in milk for males [37]. Fernández-Tuñas et al. found no significant association between HM composition or volume and infant sex or maternal age [38].

HM from mothers with no formal education showed lower levels of certain ceramides, diglycerides, phosphatidylcholines, sphingomyelins, and triglycerides. Higher household income was linked to higher levels of several amino acids, small molecules, and some lipid species. Socioeconomic factors such as education and income have been shown to influence HM composition. For example, mothers with greater household wealth had lower total saturated and polyunsaturated fatty acids but higher levels of specific ω-3 and ω-6 PUFAs [39], supporting existent evidence that maternal diet and lifestyle, shaped by socioeconomic status, can affect the nutritional composition of HM [40].

Higher BMI negatively correlated with certain ceramides, phosphatidylcholines, biogenic amines, fatty acids, and hexosylceramides, but positively with specific triglycerides, HArg, and 3-methylhistidine. Elevated BMI was also linked to a higher omega-6 to omega-3 ratio [41] and increased short-chain acylcarnitines [12].

HM composition also differed based on previous breastfeeding experience. Milk of mothers who had breastfed before revealed higher concentrations of hippuric acid, taurine, cholesteryl esters, EPA, TMAO, p-cresol sulfate, 1-methylhistidine, and triacylglycerols. The previous experience of breastfeeding might alter HM composition due to permanent alterations in the functioning of the mammary glands and maternal metabolism, which could influence lipid synthesis and amino acid-related metabolites. Mother parity and previous lactation experience have been found to affect the composition of HM. As an illustration, untargeted metabolomics profiling has demonstrated considerable variations in the metabolic profiles of mature human milk across maternal parity history with hundreds of metabolites varying according to the number of prior births [42].

### 4.4. Key Findings of Enrichment Analysis

Certain metabolic pathways were strongly linked to the child’s age. These included the production of amino acids like valine, leucine, isoleucine, arginine, phenylalanine, tyrosine, and tryptophan. Valine, leucine, and isoleucine are known as branched-chain amino acids (BCAAs), essential for building proteins and are especially important for rapid growth and development in babies [43].

Further, some pathways were linked to the mother’s BMI, such as glutathione metabolism, glycine, serine and threonine metabolism, and arginine and proline metabolism. Glutathione metabolism was strongly enriched, suggesting it plays a key role in helping infants develop a strong immune system and protecting them from oxidative stress early in life. The enrichment of glycine, serine, and threonine metabolism shows the importance of breast milk in supporting the baby’s immune system, gut health, and brain development [44]. However, these pathways’ enrichment results are emphasized as annotation-based clustering of metabolites rather than evidence of active metabolic processes.

### 4.5. Limitations and Strengths

Despite generating novel and extensive data, our study has several limitations. This analysis uses targeted metabolomics results from HM samples collected from mother–infant dyads enrolled at three lactation stages (1-, 3- and 6-months). This study employed a cross-sectional design with different mother–infant dyads represented in each age group. The composition of HM evolves across the course of lactation and is influenced by hormonal and physiological factors; therefore, longitudinal studies are needed to capture these dynamic patterns more comprehensively. Measurement of the variation was high across sample, suggesting the lack of precision and reproducibility thus effecting the clinical interpretation of the statistical analysis. Measurement of the variation was high across samples, suggesting the impact of hormonal and physiological factors; thus, longitudinal analysis is required to measure these dynamic patterns. Also, lacking standardization of the samples in terms of foremilk/hindmilk ratio, time of the day or time since last feeding, represents an additional limitation of this study.

Maternal dietary intake was not assessed, which restricts our ability to evaluate how variations in diet may influence the HM metabolite profile. Also, this study did not account for preterm birth and maternal health complications which may influence HM composition. Moreover, a very minor part of study cohort had high education, affecting the generalizability of the findings. Diurnal variation in HM macronutrients and possibly metabolites has been demonstrated in prior studies; however, the time of day at which milk samples were collected were not controlled. In the statistical analysis, although many associations reached statistical significance after FDR correction, correlation coefficients were generally modest in magnitude (r ≈ 0.1–0.3); thus, findings were interpreted as exploratory and pattern-based rather than as evidence of strong individual metabolite effects.

Despite these limitations, our study offers several strengths. The novelty of this study is that it is the first comprehensive overview of Biocrates MxP^®^ Quant 500 kit for targeted metabolic profiling of HM in Pakistan. It provides a standard for targeted metabolomics profiling in limited resource settings and highlights a novel direction for HM metabolome association with respect to maternal characteristics, adding to the existing literature while prompting further investigation. In comparison to previous studies, this study analyzed a larger sample, indicating methodological robustness. It provides a foundation for application in interventional studies and for comparison of HM composition across different demographic and clinical characteristics. By providing the pathway and enrichment analysis, this study expands the HM field beyond the association of demographic factors. The study highlights the need for future studies incorporating comprehensive maternal dietary data and a broader participant spectrum to better understand the determinants of HM composition. Considering the diurnal variation, future studies should consider the time-of-day effects when sampling and longitudinal sampling throughout lactation to better understand temporal variation in the HM metabolome among individuals. Likewise, further studies on larger and more mixed populations will assist in corroborating these results as well as enhance externalization. Moreover, the combination of metabolomic findings with infant development and health outcomes can allow us to learn more about the biological meaning of the most significant metabolites detected in this study.

## 5. Conclusions

The study findings include an investigation of all possible maternal and environmental factors to examine the confounding effect. A total of 440 metabolites were quantified, with lipids, particularly triglycerides, phosphatidylcholines, and sphingolipids, representing the predominant classes. Infant age showed the strongest association with the HM metabolome, with substantial changes observed across acylcarnitines, phosphatidylcholines, ceramides, sphingomyelins, and triglycerides. Maternal age was associated with a smaller but distinct subset of metabolites, primarily involving cholesteryl esters, sphingomyelins, triglycerides, and acylcarnitines, while infant gender showed limited associations. The enrichment of metabolites annotated to amino acid and antioxidant-related pathways highlights the coordinated representation of nutritionally relevant compounds. Future studies should focus on longitudinal mapping with assessment of maternal dietary patterns for further insight into these findings.

## Figures and Tables

**Table 1:** Participant characteristics with comparison to one-month, three-month, and six-month age groups (*n* = 267). Data is represented in mean (SD) or n (%).

Variables	Total (*n* = 267)
Mother Age (years)	25.4 (5.4)
Mother Education	
Higher Education	4 (1.5%)
Intermediate	6 (2.3%)
Middle	23 (8.6%)
No Formal Education	126 (47.19%)
Other, Specify	4 (1.5%)
Primary	70 (26.3%)
Secondary	34 (12.8%)
Mother BMI (kg/m^2^)	23.21 (5.09)
Child Gender	
Female	129 (48.5%)
Male	138 (51.68%)
Child Age (Month)	3.365 (1.908)
Mode of Delivery	
Vaginal	180 (67.41%)
Cesarean	55 (20.6%)
Frequency of Breastfeeding	
2 hourly	217 (81.27%)
4 hourly	18 (6.74%)
Previously Breastfeed	
Yes	220 (82.39%)
No	47 (17.60%)
Monthly Household Income (PKR)	32,156.3 (18,595.53)

**Table 2:** Proportion of small molecules and lipids in study HM sample.

Type	Metabolite Class	N (%)
Small Molecules	Amine oxides	1 (1.33%)
	Amino acid related	16 (21.33%)
	Amino acids	20 (26.67%)
	Bile acids	1 (1.33%)
	Biogenic amines	5 (6.67%)
	Carbohydrates and related	1 (1.33%)
	Carboxylic acids	3 (4.00%)
	Cresols	1 (1.33%)
	Fatty acids	11 (14.67%)
	Hexosylceramides	13 (17.33%)
	Indoles and derivatives	2 (2.67%)
	Vitamins and cofactors	1 (1.33%)
Lipids	Acylcarnitines	6 (1.64%)
	Ceramides	20 (5.48%)
	Cholesteryl esters	8 (2.19%)
	Diglycerides	31 (8.49%)
	Dihexosyl-ceramides	6 (1.64%)
	Lysophosphatidyl-cholines	7 (1.92%)
	Phosphatidyl-cholines	52 (14.25%)
	Sphingomyelins	15 (4.11%)
	Triglycerides	218 (59.73%)
	Trihexosyl-ceramides	2 (0.55%)

**Table 3:** Median and interquartile (IQR) concentrations [µM)] of HM metabolite classes among three age groups of children, 1-month, 3-months, and 6-months, and FDR *p*-value.

Metabolite Class	1-Month (*n* = 105)	3-Months (*n* = 106)	6-Months (*n* = 55)	FDR *p*-Value	Percentage < LOD
Acylcarnitines	1.66 [0.77–7.3]	1.46 [0.74–5.63]	1.42 [0.81–5.2]	0.31	7129/10,680 (66.75%)
Amine oxides	1.21 [0.68–1.91]	0.9 [0.6–1.39]	0.64 [0.39–0.93]	<0.01	7/267 (2.62%)
Amino acid related	1.25 [0.38–10.3]	1.23 [0.35–11.4]	1.04 [0.28–8.49]	0.01	3103/8010 (38.73%)
Amino acids	32.6 [13.9–92.55]	33.25 [14.5–108]	21 [9.48–81.92]	<0.01	32/5340 (0.59%)
Biogenic amines	1.09 [0.49–2.49]	0.9 [0.46–2.2]	0.74 [0.36–2.37]	0.04	1075/2403 (44.73%)
Carbohydrates and related	1543 [1083–2018]	1648 [1153–1989]	1407 [940–1696.5]	0.06	0/267 (0%)
Carboxylic acids	9.57 [6.16–14.15]	7.99 [4.5–12.1]	5.68 [2.99–7.9]	<0.01	711/1869 (38.04%)
Ceramides	0.21 [0.09–0.48]	0.21 [0.09–0.47]	0.24 [0.1–0.61]	<0.01	934/7476 (12.49%)
Cholesteryl esters	0.66 [0.28–1.65]	0.65 [0.23–1.42]	0.88 [0.33–2.03]	<0.01	2754/5874 (46.88%)
Cresols	2.98 [1.12–5.76]	2.66 [1.37–4.3]	1.95 [1.2–3.13]	0.1	3/267 (1.12%)
Diglycerides	2.75 [0.57–18.85]	2.34 [0.53–19]	3.73 [0.87–33.5]	<0.01	2636/11,748 (22.43%)
Dihexosyl-ceramides	0.31 [0.2–0.49]	0.33 [0.2–0.56]	0.51 [0.3–0.83]	<0.01	584/2403 (24.3%)
Fatty acids	199.5 [65.43–2052.75]	158.5 [53.48–2079.25]	196.5 [65.1–2344.25]	0.25	328/3204 (10.23%)
Hexosylceramides	0.48 [0.07–1.3]	0.4 [0.07–1.08]	0.54 [0.1–1.57]	<0.01	501/5073 (9.87%)
Indoles and derivatives	0.23 [0.09–0.52]	0.29 [0.13–0.5]	0.23 [0.12–0.41]	0.25	462/1068 (43.25%)
Lysophosphatidyl-cholines	1.04 [0.32–2.69]	0.84 [0.26–2.22]	1.26 [0.38–2.85]	<0.01	820/3204 (25.59%)
Phosphatidyl-cholines	0.22 [0.06–0.69]	0.18 [0.05–0.56]	0.25 [0.07–0.79]	<0.01	5311/20,826 (25.5%)
Sphingomyelins	0.23 [0.1–2.98]	0.18 [0.07–2.47]	0.21 [0.08–2.75]	0	8/4005 (0.19%)
Triglycerides	24.65 [5.78–112]	21.5 [4.98–101]	31.1 [7.88–155]	0	4141/64,614 (6.40%)
Trihexosyl-ceramides	0.13 [0.08–0.21]	0.11 [0.06–0.2]	0.15 [0.09–0.26]	0	813/1602 (50.74%)
Vitamins and cofactors	195 [111–286]	210 [135.25–372.75]	188 [112.5–264]	0.27	0/267 (0%)

## Data Availability

The datasets presented in this article are not readily available because the data are part of an ongoing study. Requests to access the datasets should be directed to the corresponding author.

## Supplementary Materials

The following supporting information can be downloaded at: https://www.mdpi.com/article/10.3390/metabo16030162/s1, Table S1: Biocrates MxP^®^ QUANT metabolite Details, Table S2: Correlation matrix showing significant associations between human milk (HM) metabolites and (A) maternal age, (B) child age, (C) child gender, (D) delivery type (E) previous breastfeeding experience, (F) maternal body mass index (BMI), (G) household income, and (H) maternal education; Table S3: Multivariable regression outcome showing significant associations between HM metabolites and (A) maternal age, (B) child age, (C) child gender, (D) delivery type (E) previous breastfeeding experience, (F) maternal body mass index (BMI), (G) household income, and (H) maternal education; Table S4: Sensitive analysis outcome showing significant associations between HM metabolites and (A) maternal age, (B) child age, (C) child gender, (D) delivery type (E) previous breastfeeding experience, (F) maternal body mass index (BMI), (G) household income, and (H) maternal education, Figure S1: Triglycerides composition in 1-month, 3-month and 6-month age grouped children. Data are expressed as median concentration. * *p*_value < 0.05, ** *p*_value < 0.01, *** *p*_value, Figure S2: Phosphatidyl-choline composition in 1-month, 3-month and 6-month age grouped children. Data are expressed as median concentration. * *p*_value < 0.05, ** *p*_value < 0.01, *** *p*_value < 0.001.s.

## Abbreviations

The following abbreviations are used in this manuscript:

HM	Human milk
HPLC	High-performance liquid chromatography
LC–MS/MS	Liquid chromatography-tandem mass spectrometry
SM	Small molecule
ERC	Ethical review committee
ID	Identification
PHC	Primary healthcare center
MINE	Maternal and environmental Impact assessment on Neurodevelopment in Early childhood years
PRISMA	The Pregnancy Risk Infant Surveillance and Measurement Alliance
