# Supplementary material for: Association Between Human Milk-Targeted Metabolites and Maternal Characteristics: Targeted Metabolomic Profiling of Human Milk in Low-Income Settings
Source: Metabolites. 2026 Feb 28;16(3):162. doi: 10.3390/metabo16030162 (PMC13028068; doi:10.3390/metabo16030162)
Supplement: Supplementary file 1 [file metabolites-16-00162-s001.zip › Questionniare (2).pdf]

MINE Study Visit 1

CHILD'S STUDY ID: بچے کی مطالعہ کی شناخت

M	I	N	E	-	P	K	-				

**Interview/Assessment Dates انٹرویو/تشخیص کی تاریخ**		
1.	**Interview/Assessment Date of 1st Visit** پہلا وزٹ انٹرویو/تشخیص کی تاریخ	__ __ / __ __ / __ __ __ __ (DD / MM / YYYY) (سال/مہینہ/دن)
2.	**Screening Location/Site** اسکریننگ لوکیشن/سائٹ	**1-** Ibrahim Hyderi ابراھیم حیدری<br>**2-** Rehri Goth ریڑھی گوٹھ
3.	**Child VR-ID** بچے کی وی -آر آئی ڈی	______________________________
4.	**Mother VR-ID (ARC Study)** ماں کی وی-آر آئی ڈی (اے آر سی اسٹیڈی)	______________________________
5.	**Staff ID/Name** اسٹاف آئی ڈی	______________________________
6.	**Outcome of Visit :** وزٹ کا نتیجہ	
	**1)** Complete مکمل<br>**2)** The eligible respondent was not home at the time of the visit (Revisit – Skip to Form Completion)<br>اہل جواب دہندہ دورے کے وقت گھر پر نہیں تھا (دوبارہ ملاحظہ کریں - فارم کی تکمیل کے سیکشن پر جائیں)<br>**3)** The eligible respondent refused to give an interview (Skip to Form Completion)<br>اہل جواب دہندہ نے انٹرویو دینے سے انکار کر دیا (فارم مکمل کرنے کے سیکشن پر جائیں)<br>**4)** No family member was available at home (Skip to Form Completion<br>گھر پر خاندان کا کوئی رکن دستیاب نہیں تھا (دوبارہ ملاحظہ کریں - فارم کی تکمیل کے سیکشن پر جائیں)<br>**5)** Migrated (Skip to Form Completion)<br>ہجرت شدہ (فارم مکمل کرنے والے حصے پر جائیں)	

	**Child Details بچے کی معلومات**			
7.	**Name of Child** بچے کانام	______________________________		
8.	**Father Name** والدکانام	______________________	9.	Age<br>عمر|_____|_____|Yrs.سال
10.	**Mother Name** والدہ کانام	______________________	11.	Age<br>عمر|_____|_____|Yrs.سال
12.	**Address** پتہ			
	House #<br>گھر______________________________<br>Sector/Area ایریا / سیکٹر<br>______________________________<br>Landmark لینڈ مارک<br>______________________________			

13.	**Gender** جنس	○ Male مرد<br>○ Female عورت<br>○ Other دیگر
14.	**Birth Order** ترتیب پیدائش	|_____|_____|
15.	**Date of Birth** تاریخ پیدائش	__ __ / __ __ / __ __ __ __<br>(DD / MM / YYYY) (سال/مہینہ/دن)
16.	**Age (in months)** (عمر (مہینوں میں	|_____|_____|

	**Child Physical Health بچے کی جسمانی صحت**				
17.	**Weight** وزن	_________kg<br>کلوگرام	**18.**	**Height** اونچائی	_________cm

19.	**MUAC** ایم یو اے سی	________cm	**20.**	**Head Circumference** سر کا دائرہ	________cm
21.	**Birth Weight** پیدائشی وزن	________kg<br>کلوگرام	**22.**	**Birth Length** پیدائش کی لمبائی	________cm

Any Illness in Last 7 Days پچھلے 7 دنوں میں کوئی بھی بیماری

23.	Has the child been sick with any of the illness below in the past 7 Days?<br>کیا بچہ پچھلے 7 دنوں میں نیچے دی گئی کسی بیماری سے بیمار ہوا ہے؟	☐ Yesہاں<br>☐Noنہیں<br>☐ Don't know معلوم نہیں	If no, skip to question 27<br>اگر نہیں تو سوال 27 پر جائیں۔<br><br>If yes, go to the next question<br>اگر ہاں، تو اگلے سوال پر جائیں۔
24.	a. ☐ Fever / Hotness of body<br>بخار / جسم کی گرمی	b. ☐ Seizures دورے	c. ☐ Vomiting الٹی
	**d.** ☐ Difficulty breathing<br>سانس لینے میں دشواری	**e.** ☐ Diarrhoea اسہال	**f.** ☐ Altered consciousness بدلا ہوا شعور
	**g.** ☐ Lethargy سستی	**h.** ☐ Moaning/ grunting<br>کراہنا/ کراہنا	**i.** ☐ Poor Feeding ناقص خوراک
	**j.** ☐ Irritable and crying<br>چڑچڑا اور رونا	**k.** ☐ Any Other<br>کسی بھی دیگر	

Any medication Used for the above Illnesses مندرجہ بالا بیماریوں کے لیے استعمال ہونے والی کوئی بھی دوا

25.	☐ Yes ہاں	☐ No نہیں	☐ If yes, Skip to question no. 26<br>اگر ہاں تو سوال نمبر 26 پر جائیں۔<br>☐ If no, skip to question no. 27<br>اگر نہیں، تو سوال نمبر27 پر جائیں

Child Medication Use بچوں کی دوائیوں کا استعمال

Drugs taken: (include medication used in hospital as well as medication for use after discharge)
لی گئی دوائیں: (اسپتال میں استعمال ہونے والی دوائیوں کے ساتھ ساتھ ڈسچارج کے بعد استعمال کی دوائیں بھی شامل ہیں)

**26.**	**Name of drug**<br>دوا کا نام	**Route**<br>**(i.e. I.M, I.V., Oral, Rectal)**	**Dose amount**<br>**(i.e. 50, 100)**	**Dose units**<br>**(i.e., µg/ml, ml, mg)**	**Frequency of dose**<br>**(i.e., once/day, 3 times/day)**	**No of days of treatment**<br>**(i.e., 3 days)**
I.						
II.						
III.						
IV.						

27.	**Hospitalization History in the Last Three Months**<br>پچھلے تین مہینوں میں ہسپتال میں داخل ہونے کی تاریخ	**1-** Yes ہاں<br>Specify the no. of times child was hospitalized in last three months |___|___| نمبر کی وضاحت کریں۔ پچھلے تین مہینوں میں کتنی بار بچہ ہسپتال میں داخل ہوا<br>**2-** No → *Skip to question 29*<br>نہیں،سوال نمبر 29 پر جائیں۔

28.	Reason for hospitalization<br>ہسپتال میں داخل ہونے کی وجہ	

Vaccination History ویکسی نیشن کی تاریخ

29.	**Has the child been vaccinated?**<br>کیا بچے کو ویکسین لگائی گئی ہے؟	☐Yes, skip to question 31 -ہاں، سوال 31 پر جائیں<br>☐ No, skip to question 30 -نہیں، سوال30 پر جائیں<br>☐ Unable to recall, skip to question 29<br>یاد کرنے سے قاصر، سوال 29 پر جائیں-
30.	**If No, Tick any one of the responses**<br>اگر نہیں تو جوابات میں سے کسی ایک پر نشان لگائیں-	☐ Unaware of the need for vaccine<br>ویکسین کی ضرورت سے بے خبر<br>☐ Concerns regarding the myths about the vaccine<br>ویکسین کے بارے میں خرافات کے حوالے سے خدشات<br>☐ Scared of the side effects<br>ضمنی اثرات سے خوفزدہ<br>☐ Place of immunization too far<br>حفاظتی ٹیکوں کی جگہ بہت دور<br>☐ Unaffordable traveling cost health care facility<br>ناقابل برداشت سفری لاگت صحت کی دیکھ بھال کی سہولت<br>☐ Fear of multiple vaccinations<br>متعدد ویکسین کا خوف<br>☐ Parents/guardians too busy<br>والدین/سرپرست بہت مصروف ہیں-<br>Others (Specify) (وضاحت کریں) دیگر ------------------------------

**31.**	**Vaccine** ویکسین	**Received vaccine?** (Check one) ویکسین مل گئی؟ (ایک چیک کریں)			**Source of report** (Indicate source for each vaccine) رپورٹ کا زریعہ (ہر ویکسین کے ذریعہ کی نشاندہی کریں)		**Date** (Fill completely) تاریخ (مکمل طور پر بھریں)		
		1= Yes ہاں	2= No نہیں	3= DK معلوم نہیں	1= Card کارڈ	2= Verbal زبانی	DD دن	MM مہینہ	YY سال
I.	**BCG (Vaccination for TB)** بی سی جی (ٹی بی کے لیے ویکسی نیشن)								
II.	**OPV-0 (Polio drops at birth)** او پی وی-0(پیدائش کے وقت پولیو کے قطرے)								
III.	**Hepatitis-B Vaccine (at birth)** ہیپاٹائٹس بی ویکسین (پیدائش کے وقت)								
IV.	**OPV-1 (Polio drops at 6 weeks)** او پی وی-1(پولیو کے قطرے 6) ہفتوں میں								
V.	**Penta-1 (Pentavalent 1 at 6 weeks)** پینٹا -1 (پینٹا ویلنٹ 1 6 ہفتوں میں)								
VI.	**PCV-1 (Pneumococcal Conjugate at 6 weeks)** پی سی وی -1(6 ہفتوں میں نیوموکوکل کنجوگیٹ)								
VII.	**RV-1 (Rotavirus vaccine at 6 weeks)**								

	آر وی -1(6 ہفتوں میں روٹا وائرس ویکسین)								
VIII.	**OPV-2 (Polio drops at 10 weeks)**<br>او پی وی-2(10 ہفتوں میں پولیو کے قطرے)								
IX.	**Penta-2 (Pentavalent 2 at 10 weeks)**<br>پینٹا -2 (پینٹا ویلنٹ -2 10 ہفتوں میں)								
X.	**PCV-2 (at 10 weeks)**<br>پی سی وی -2(10 ہفتوں میں)								
XI.	**RV-2 (Rotavirus vaccine 2) (at 10 weeks)**<br>آر وی -2(10ہفتوں میں روٹا وائرس ویکسین)								
XII.	**OPV-3 (Polio drops) (at 14 weeks)**<br>او پی وی-3(14ہفتوں میں پولیو کے قطرے)								
XIII.	**Penta-3 (at 14 weeks)**<br>پینٹا -3 (پینٹا ویلنٹ -3 14 ہفتوں میں)								
XIV.	**PCV-3 (at 14 weeks)**<br>پی سی وی -3(14 ہفتوں میں)								
XV.	**IPV1 (at 14 weeks)**<br>آئی پی وی -2(14 ہفتوں میں)								

Maternal Details زچگی کی تفصیلات

**32. Mother Physical Health ماں کی جسمانی صحت**			
	Weight وزن _______ Kgs کلوگرام	Height اونچائی __ Cm	BMIبی ایم آئی __________
**33. Education تعلیم**			
	☐ No formal education کوئی رسمی تعلیم نہیں	☐ Primary پرائمری	☐ Middleمڈل
	☐ Secondary ثانوی	☐ Intermediate انٹرمیڈیٹ	☐ Higher Education اعلی تعلیم
	☐ Other, specify دیگر کی وضاحت _______________		
**34. Breastfeeding History دودھ پلانے کی ہسٹری**			
	Feeding of colostrum کولسٹرم کا کھانا کھلانا	☐ Yes ہاں ☐ No نہیں ☐ Unknown معلوم نہیں	
	Breastfeeding frequency<br>دودھ پلانے کی تعدد	☐ 2 hourly 2گھنٹہ ☐ 4 hourly 4گھنٹہ	
	How early was colostrum/breastfeeding initiated<br>کولسٹرم/بریسٹ فیڈنگ کتنی جلدی شروع کی گئی تھی-	☐ Within two hours birth پیدائش کے اندر 2 گھنٹے<br>☐ Within 4-8 hours اندر 4-8گھنٹے کے<br>☐ Within 8-24 hours اندر 8-24گھنٹے کے	
	How many children has been breastfed before this infant<br>اس شیر خوار سے پہلے کتنے بچوں کو دودھ پلایا گیا ہے؟	☐ None, first child, کوئی نہیں، پہلا بچہ ☐ 1 ☐ 2 ☐ 3<br>☐ More than 3 3سے زیادہ	

	Is mother taking any multivitamins<br>کیا ماں کوئی ملٹی وٹامن لے رہی ہے؟	☐ Yes ہاں ☐No نہیں	☐ If yes, please specify<br>اگر ہاں، دیگر وضاحت کریں<br>________________
**35. Monthly Income ماہانہ آمدنی**			
	☐ Employed, skip to question 36- ملازم، سوال 36 پر جائیں<br>☐Unemployed, skip to question 38- بے روزگار، سوال 38 پر جائیں		
36.	Mother's Occupation والدہ کا پیشہ	Business کاروبار ---------------------------- 1<br>Farming کاشتکاری -----------------------------2<br>General Labour جنرل لیبر-------------------- 3<br>Govt. Service حکومت سروس -------------------4<br>Housewife گھریلو خاتون------------------------5<br>Landlord مالک مکان ----------------------------6<br>Shop Keeper شاپ کیپر -------------------------7<br>Student طالب علم --------------------------------8<br>Teacher استاد-------------------------------------9<br>Vendor وینڈر----------------------------------- 10<br>Unemployed بے روزگار --------------------- 11<br>Retired ریٹائرڈ --------------------------------- 12<br>Private Service پرائیویٹ سروس -------------- 13<br>Factory Worker فیکٹری ورکر----------------- 14<br>Others specify):(وضاحت کریں) دیگر ----------	
37.	☐ Monthly income |____|____|____|____|____|____|		
**38. Medical/Clinical History طبی/کلینکل ہسٹری**			
	**Chronic hypertension**<br>دائمی ہائی بلڈ پریشر	☐ Yes ہاں ☐No نہیں ☐ Don't know معلوم نہیں	
	**Cardiac diseases**<br>دل کے امراض	☐ Yes ہاں ☐No نہیں ☐ Don't know معلوم نہیں	
	**Anaemia**<br>خون کی کمی	☐ Yes ہاں ☐No نہیں ☐ Don't know معلوم نہیں	
	**Diabetes mellitus**<br>ذیابیطس	☐ Yes ہاں ☐No نہیں ☐ Don't know معلوم نہیں	
	**Any other Illness**<br>کوئی دوسری بیماری	☐ Yes ہاں ☐No نہیں ☐ Don't know معلوم نہیں	
**Psychosocial/Mental Health History نفسیاتی/ذہنی صحت کی ہسٹری**			
39.	**Postpartum Depression (EPDS)**	Total score اسکورٹوٹل |____|____| Specify واضح کریں → **1-** Possible depression ممکنہ ڈپریشن<br>2- No depression کوئی ڈپریشن نہیں	
40.	**Intimate Partner Violence (HITS)**	Total score ٹوٹل اسکور |____|____| Specify واضح کریں → **1-** Positive مثبت **2-** Negative منفی	
41.	**Parenting Stress Index (PSI-LV)**	Total score ٹوٹل اسکور |____|____| Specify واضح کریں → **1-** Higherاعلی **2-** Averageاوسط<br>**3-** Lower نیچے	
42.	**Maternal Autonomy (MAI)**	Total score ٹوٹل اسکور |____|____| Specify واضح کریں → **1-** Higherاعلی **2-** Averageاوسط<br>**3-** Lower نیچے	
43.	**Hours of sleep taking per day**<br>ایک دن میں کتنے گھنٹہ نیند لیتے ہیں	Total hours ٹوٹل اسکور |____|____|	
44.	**Any other significant diagnosed mental health history**<br>دماغی صحت کی کوئی دوسری اہم تشخیص شدہ تاریخ	**1-** Yes ہاں→ Specify واضح کریں ____________________<br>**2-** No نہیں	

45.	**If yes, is she taking any medicine**<br>اگر ہاں، تو کیا وہ کوئی دوا لے رہی ہے؟	☐ Yes ہاں ☐No نہیں
**Any medication Used for the above Illnesses مندرجہ بالا بیماریوں کے لیے استعمال ہونے والی کوئی بھی دوا**		
46.	☐ Yes ہاں	☐ Noنہیں
	☐ If yes, go to questions no. 47 -اگر ہاں تو سوال نمبر 47 پر جائیں<br>☐ If no, skip to question no. 48 -اگر نہیں تو سوال نمبر 48 پر جائیں	

Mother Medication Use والدہ کی دوائی کا استعمال

Drugs taken: (include medication used in hospital as well as medication for use after discharge)
لی گئی دوائیں: (اسپتال میں استعمال ہونے والی دوائیوں کے ساتھ ساتھ ڈسچارج کے بعد استعمال کی دوائیں بھی شامل ہیں)

47.	**Name of drug** دوا کا نام	**Route (i.e. I.M, I.V., Oral, Rectal)**	**Dose amount (i.e. 50, 100)**	**Dose units (i.e., µg/ml, ml, mg)**	**Frequency of dose (i.e., once/day, 3 times/day)**	**No of days of treatment (i.e., 3 days)**
I.						
II.						
III.						
IV.						

Pregnancy Details حمل کی تفصیلات

48.	Are you currently pregnant?<br>کیا آپ فی الحال حاملہ ہیں؟	1. Yes ہاں ------------------------------------<br>2. Noنہیں --------------------------------------

49. Details of Pregnancies حمل کی تفصیلات

	**50. Date of outcome** نتائج کی تاریخ	**51. Outcome** If 1,2,5→ Skip to next pregnancy<br>نتیجہ<br>اگر 1,2,5اگلے حمل پر جائیں۔	**52. Current Status of child * If**<br>بچے کی موجودہ حالت*<br>اگر	**53. Age of alive child** زندہ بچے کی عمر			**54. Age at time of death** وفات کے وقت عمر		
	**DD/MM/YYYY** دن/مہینہ/اس ال			**Years** سال	**Months** مہینہ	**Days** دن	**Years** سال	**Months** مہینہ	**Days** دن
**1.**									
**2.**									
**3.**									
**4.**									
**5.**									
**6.**									
**7.**									
**8.**									
**9.**									
**10**									
**11**									

50.	**Options for 50**<br>50کے لیے اختیارات	**1=Miscarriages** اسقاط حمل<br>**2=Still Birth** ابھی تک پیدائش	**Options for 51**	**1= Alive**<br>زندہ

		**3=Live Birth** زندہ پیدائش<br>**4=Twin Birth Alive** زندہ جڑواں پیدائش<br>**5= Twin Still Birth** جڑواں اب بھی پیدائش<br>**6= One Still Birth & one Live Birth** ایک اب بھی پیدائش اور ایک زندہ پیدائش	51کے لیے اختیارات	**2= Died**<br>وفات پائی

**Substance Abuse for Mother**		
52.	☐ Cigarette سگریٹ<br>☐Yes ہاں<br>**If Yes →** How often? اگر ہاں، کتنی بار؟<br>**1)** Daily روزانہ<br>**2)** Weekly ہفتہ وار<br>**3)** < a month <ایک مہینہ<br>☐ No نہیں<br>☐Only outside صرف باہر	☐ Tobacco تمباکو<br>☐Yes ہاں<br>**If Yes →** How often? اگر ہاں، کتنی بار؟<br>**1)** Daily روزانہ<br>**2)** Weekly ہفتہ وار<br>**3)** < a month <ایک مہینہ<br>☐ No نہیں<br>☐Only outside صرف باہر
53.	☐ Alcohol الکوہل<br>☐Yes ہاں<br>**If Yes →** How often? اگر ہاں، کتنی بار؟<br>**1)** Daily روزانہ<br>**2)** Weekly ہفتہ وار<br>**3)** < a month <ایک مہینہ<br>☐ No نہیں<br>☐Only outside صرف باہر	☐ Betel Nets چھالیہ<br>☐Yes ہاں<br>**If Yes →** How often? اگر ہاں، کتنی بار؟<br>**1)** Daily روزانہ<br>**2)** Weekly ہفتہ وار<br>**3)** < a month <ایک مہینہ<br>☐ No نہیں<br>☐Only outside صرف باہر
54.	☐ Chaali چلی<br>☐Yes ہاں<br>**If Yes →** How often? اگر ہاں، کتنی بار؟<br>**1)** Daily روزانہ<br>**2)** Weekly ہفتہ وار<br>**3)** < a month <ایک مہینہ<br>☐ No نہیں<br>☐Only outside صرف باہر	☐ Gutka گٹکا<br>☐Yes ہاں<br>**If Yes →** How often? اگر ہاں، کتنی بار؟<br>**1)** Daily روزانہ<br>**2)** Weekly ہفتہ وار<br>**3)** < a month <ایک مہینہ<br>☐ No نہیں<br>☐Only outside صرف باہر
55.	☐ Any other, please specify<br>کوئی اور، براہ کرم وضاحت کریں	

Reynolds Intellectual Assessment Scales (RIAS) (For Father)
رینالڈز انٹلیکچوئل اسسمنٹ اسکیلز (RIAS) (والد کے لیے)

**56.**	**Odd-Item Out (OIO)** اوڈ آئٹم آوٹ	
**57.**	**What's Missing (WHM)** کیا غائب ہے	
**58.**	**Nonverbal Intelligence Index (NIX)** غیر زبانی انٹیلی جنس انڈیکس	

Farther Details والد کی تفصیلات

**59.**	**Education تعلیم**		
	☐ No formal education کوئی رسمی تعلیم نہیں	☐ Primary پرائمری	☐ Middleمڈل
	☐ Secondary ثانوی	☐ Intermediate انٹرمیڈیٹ	☐ Higher Education اعلی تعلیم
	☐ Other, specify دیگر کی وضاحت _______________		

**60. Medical/Clinical History** طبی/کلینکل ہسٹری		
	**a. Chronic hypertension**<br>دائمی ہائی بلڈ پریشر	☐ Yes ہاں ☐No نہیں ☐ Don't know معلوم نہیں
	**b. Cardiac disease**<br>دل کے امراض	☐ Yes ہاں ☐No نہیں ☐ Don't know معلوم نہیں
	**c.** Any significant diagnosed mental health history	☐ Yes ہاں ☐No نہیں ☐ Don't know معلوم نہیں

	دماغی صحت کی کوئی بھی اہم<br>تشخیص شدہ ہسٹری	
	d. **Any other** کوئی دوسرا	**1)** Yes → Specify........... **1**- Previous **2**- Recent<br>No ☐ Don't know
**Any medication Used for the above Illnesses** مندرجہ بالا بیماریوں کے لیے استعمال ہونے والی کوئی بھی دوا		
61.	Are Using any medication for any of the above illnesses<br>مندرجہ بالا بیماریوں میں سے کسی کے لیے کوئی دوا استعمال کر رہے ہیں۔	☐ If yes, go to questions no. 62<br>اگر ہاں تو سوال نمبر 62 پر جائیں۔<br>☐ If no, skip to question no. 63<br>اگر نہیں تو سوال نمبر 63 پر جائیں۔

Father Medication Use والد کی دوائیوں کا استعمال

Drugs taken: (include medication used in hospital as well as medication for use after discharge)
لی گئی دوائیں: (اسپتال میں استعمال ہونے والی دوائیوں کے ساتھ ساتھ ڈسچارج کے بعد استعمال کی دوائیں بھی شامل ہیں)

62.	**Name of drug**<br>دوا کا نام	**Route**<br>**(i.e. I.M, I.V., Oral, Rectal)**	**Dose amount**<br>**(i.e. 50, 100)**	**Dose units**<br>**(i.e., µg/ml, ml, mg)**	**Frequency of dose**<br>**(i.e., once/day, 3 times/day)**	**No of days of treatment**<br>**(i.e., 3 days)**
I.						
II.						
III.						
IV.						

**Substance Abuse for Father**		
63.	☐ Cigarette سگریٹ<br>☐Yes ہاں<br>**If Yes →** How often? اگر ہاں، کتنی بار؟<br>**1)** Daily روزانہ<br>**2)** Weekly ہفتہ وار<br>**3)** < a month <ایک مہینہ<br>☐ No نہیں<br>☐Only outside صرف باہر	☐ Tobacco تمباکو<br>☐Yes ہاں<br>**If Yes →** How often? اگر ہاں، کتنی بار؟<br>**1)** Daily روزانہ<br>**2)** Weekly ہفتہ وار<br>**3)** < a month <ایک مہینہ<br>☐ No نہیں<br>☐Only outside صرف باہر
64.	☐ Alcohol الکوہل<br>☐Yes ہاں<br>**If Yes →** How often? اگر ہاں، کتنی بار؟<br>**1)** Daily روزانہ<br>**2)** Weekly ہفتہ وار<br>**3)** < a month <ایک مہینہ<br>☐ No نہیں<br>☐Only outside صرف باہر	☐ Betel Nets چھالیہ<br>☐Yes ہاں<br>**If Yes →** How often? اگر ہاں، کتنی بار؟<br>**1)** Daily روزانہ<br>**2)** Weekly ہفتہ وار<br>**3)** < a month <ایک مہینہ<br>☐ No نہیں<br>☐Only outside صرف باہر
65.	☐ Chaali چلی<br>☐Yes ہاں<br>**If Yes →** How often? اگر ہاں، کتنی بار؟<br>**1)** Daily روزانہ	☐ Gutka گٹکا<br>☐Yes ہاں<br>**If Yes →** How often? اگر ہاں، کتنی بار؟<br>**1)** Daily روزانہ

	**2)** Weekly ہفتہ وار<br>**3)** < a month <ایک مہینہ<br>☐ No نہیں<br>☐Only outside صرف باہر	**2)** Weekly ہفتہ وار<br>**3)** < a month <ایک مہینہ<br>☐ No نہیں<br>☐Only outside صرف باہر
	☐ Any other, please specify<br>کوئی اور، براہ کرم وضاحت کریں	

**Profession / Job**			
66.	☐ Employed ملازم ☐Unemployed بے روزگار<br>☐ If unemployed, skip to question 69, ، اگر بے روزگار ہیں تو اگر ہاں تو سوال نمبر9 6 پرجائیں		
67.	☐ Fisherman ماہی گیر	☐ Factory worker<br>فیکٹری مزدور	☐ Skilled labour ہنر مند لیبر
	☐ Unskilled labour غیر ہنر مند مزدور	☐ Salesman سیلز مین	☐ Mason مستری
	☐ Mechanic مکینک	☐ Healthcare staff<br>صحت کی دیکھ بھال کا عملہ	☐ Small business چھوٹا کاروبار
	☐ Farming کھیتی باڑی	☐ Unemployed/ Not working<br>بے روزگار/ کام نہیں کرنا	☐ Other دیگر___________________
68.	**Monthly Income**	|____|____|____|____|____|____|	

**Sociodemographic Details سماجی آبادیاتی تفصیلات**					
People Details لوگوں کی تفصیلات					
69.	Contact No. of HH ایچ ایچ کا رابطہ نمبر	|_________________________________|			
70.	Female عورت (>19yrs)	|____|____|	71.	Adolescents نوعمر (10-19yrs)	|____|____|
72.	Male مرد (>19yrs)	|____|____|	73.	Adolescents نوعمر (M)<br>Adolescents نوعمر (F)	|____|____| |____|____|
74.	Children under 5yrs<br>5 سال سے کم عمر کے بچے	Boys: |____|____| Girls: |____|____|	75.	Total Family Members (including children)<br>خاندان کے کل ارکان (بشمول بچوں)	Male: مرد|____|____| Female: عورت|____|____|
**Room Details** کمرے کی تفصیلات					
76.	☐Total no. of rooms in house گھر میں کمروں کی کل تعداد |____|____|				
77.	☐Total no. of rooms utilized by child بچوں کے زیر استعمال کمروں کی کل تعداد |____|____|				
**Kitchen Details** باورچی خانے کی تفصیلات					
78.	What type of cook stove is mainly used for cooking in your household?<br>آپ کے گھر میں کھانا پکانے کے لیے بنیادی طور پر کس قسم کا چولہا استعمال ہوتا ہے؟	1) Electric stove الیکٹرک چولہا<br>2) Solar cooker سولر کولر<br>3) Liquefied petroleum gas/cooking gas stove<br>مائع پیٹرولیم گیس / کھانا پکانے کا چولہا<br>4) Piped natural gas stove<br>قدرتی گیس کا چولہا۔<br>5) Biogas stove / Liquid fuel stove<br>بائیو گیس کا چولہا / مائع ایندھن کا چولہا۔<br>6) Manufactured solid fuel stove<br>ٹھوس ایندھن کا چولہا تیار کیا گیا۔<br>7) Traditional solid fuel stove<br>روایتی ٹھوس ایندھن کا چولہا۔			

		8) Three stone stove/open fire<br>تین پتھر کا چولہا/کھلی آگ<br>9) No food cooked in household<br>گھر میں کھانا نہیں پکتا<br>10) Other, specify دیگر کی وضاحت ________________
79.	What type of fuel or energy source is used in this cook stove?<br>اس کھانا پکانے والے چولہے میں کس قسم کا ایندھن یا توانائی کا ذریعہ استعمال ہوتا ہے؟	1) Electricity بجلی<br>2) Solar energy شمسی توانائی<br>3) Alcohol/ethanol الکحل / ایتھنول<br>4) Gasoline/diesel پٹرول/ڈیزل<br>5) Kerosene/paraffin مٹی کا تیل / پیرافین<br>6) Coal/lignite/charcoal کوئلہ/لگنائٹ/چارکول<br>7) Wood لکڑی<br>8) Straw/shrubs/grass تنکے/جھاڑیاں/گھاس<br>9) Agricultural crop زرعی فصل<br>10) Animal dung/waste جانوروں کا گوبر / فضلہ<br>11) Processed biomass (pellets) or woodchips<br>پروسس شدہ بایوماس (چھرے) یا لکڑی کے چپس<br>12) Garbage/plastic کچرا/پلاسٹک<br>13) Sawdust لکڑی کا برادہ<br>14) Other, specify دیگر کی وضاحت________________
80.	Is cooking usually done inside the house?<br>کیا کھانا پکانا عموماً گھر کے اندر ہوتا ہے؟	1) Yes<br>2) No
81.	Do you have a separate room which is used as a kitchen?<br>کیا آپ کے پاس ایک علیحدہ کمرہ ہے جو باورچی خانے کے طور پر استعمال ہوتا ہے؟	1) Yes<br>2) No
82.	Are there windows or doors for ventilation in the room that is usually used for cooking?<br>کیا کمرے میں وینٹیلیشن کے لیے کھڑکیاں یا دروازے ہیں جو عام طور پر کھانا پکانے کے لیے استعمال ہوتے ہیں؟	1) Yes<br>2) No<br>3) N/A (outdoors)
**Toilet Details** بیت الخلاء کی تفصیلات		
83.	☐ Total no. toilets in house<br>گھر میں بیت الخلا کی کل تعداد	|____|____|
84.	☐ Total no. of people utilizing the same toilet<br>بیت الخلاء استعمال کرنے والے لوگوں کی کل تعداد	|____|____|
85.	Do you share this toilet with other households?<br>کیا اس بیت الخلاء کی سہولت کو دوسرے گھرانوں کے افراد بھی استعمال کر تےہیں	1) Yes, ہاں If Yes → Q 86 اگر ہاں تو سوال نمبر 86 پرجائیں<br>2) No نہیں
86.	How many people share this toilet?<br>کتنے گھرانےاس بیت الخلاء کی سہولت کواستعمال کرتے ہیں ؟	|____|____|
87.	Where is the toilet facility located?<br>بیت الخلا کی سہولت کہاں واقع ہے؟	1) Within the household structure گھریلو ساخت کے اندر<br>2) Neighbour's home/structure پڑوسی کا گھر / ڈھانچہ<br>3) Public toilet عوامی لیٹرین<br>4) Other (Specify) دیگر کی وضاحت<br>_________________

88.	Type of toilet<br>بیت الخلاء کی اقسام	1) Flush/pour flush to piped sewer system<br>فلش یا انڈیل کر پانی ڈالنے والا<br>2) Flush/pour flush to pit latrine<br>فلش گڑھے واے لیٹرین سے جڑاہوا<br>3) Pit latrine with slab<br>گڑھے والا لیٹرین ڈھکا ہوا<br>4) Composting toilet<br>کمپوسٹ ٹوائلٹ<br>5) Hanging toilet/hanging latrine<br>ہینگنگ ٹوائلٹ/ لٹکانے والی لیٹرین<br>6) Flush/pour flush to septic tank<br>سیپٹک ٹینک میں فلش/ڈالیں۔<br>7) Flush/pour flush to somewhere else<br>کسی اور جگہ فلش/ڈالیں۔<br>8) Ventilated improved pit latrine<br>ہوادار بہتر پٹ لیٹرین<br>9) Pit latrine without slab/open toilet<br>پٹ لیٹرین بغیر سلیب/کھلے بیت الخلا کے<br>10) Bucket toilet<br>بالٹی والا ٹوائلٹ<br>11) No facility/bush/field<br>کوئی سہولت نہیں /جھاڑیوں/زمین پر<br>12) Other(specify)دیگر وضاحت کریں<br>____________________
**House Infrastructure Details** ہاؤس انفراسٹرکچر کی تفصیلات		
89.	What is the main building material of your household's floor?<br><br>آپ کے گھر کے فرش بنانے میں کس قسم کا میٹریل استعمال کیاگیا ہے	1) Earth/sand زمین/ریت<br>2) Bricks اینٹیں<br>3) Dung گوبر<br>4) Wood planks لکڑی کے تختے<br>5) Palm/bamboo کھجور / بانس<br>6) Parquet/polished wood پارکیٹ / پالش لکڑی<br>7) Vinyl/asphalt strip ونائل/اسفالٹ کی پٹی<br>8) Marble سنگ مرمر<br>9) Ceramic tiles سیرامک ٹائلز<br>10) Cement/concrete سیمنٹ/کنکریٹ<br>11) Carpet/mats قالین/چٹائیاں<br>12) Other(specify) دیگر وضاحت______________
90.	What is the main building material of your household's roof?<br><br>آپ کے گھر کی چھت میں استعمال ہونے والا بنیادی مواد؟	1) No roof کوئی چھت نہیں<br>2) Cement سیمنٹ<br>3) Metal – iron/tin sheets دھات - لوہے / ٹن کی چادریں<br>4) Thatch/palm leaf/palm/bamboo<br>کھجور / کھجور کی پتی / کھجور / بانس<br>5) Sod grass گھاس پھوس سے بنی ہوئی چھت<br>6) Rustic mat ٹنکے کی چٹائی<br>7) Cardboard گتے سے بنی ہوئی چھت<br>8) Wood planks (rudimentary roofing)<br>لکڑی کے تختے (ابتدائی چھت)<br>9) Wood (finished roofing) لکڑی سے بنی ہوئی چھت<br>10) Calamine/cement fibre کیلامین/سیمنٹ فائبر<br>11) Ceramic tiles سرامک ٹائل<br>12) Roofing shingles چھت میں استعمال ہونے والے ٹائلز<br>13) Other(specify) (وضاحت کریں) دیگر<br>________________

91.	What is the main building material of your household's exterior wall?<br><br>آپ کے گھر کی دیوار وں میں استعمال ہونے والا بنیادی مواد؟	1) No walls کوئی دیوار نہیں<br>2) Cane/palm /trunks کھجور کے درخت /بانس<br>3) Dirt/mud مٹی/کیچڑ<br>4) Dung گوبر<br>5) Straw/grass/sod تنکے/گھاس پھوس سے بنی ہوئی چھت<br>6) Bamboo with mud بانس اور مٹی سے بنی ہوئی چھت<br>7) Stone with mud کیچڑ کے ساتھ پتھر<br>8) Uncovered adobe (clay bricks dried in sun) بے نقاب ایڈوب (دھوپ میں خشک مٹی کی اینٹیں)<br>9) Covered adobe (clay bricks dried in sun with protective covering such as a thin layer of cement)<br>10) Plywood لکڑی کی پلائی<br>11) Cardboard گتے<br>12) Reused wood استعمال کی ہوئی لکڑی<br>13) Cement سیمنٹ<br>14) Stone with lime/cement پتھر چونا/سمنٹ کے ساتھ<br>15) Bricks اینٹیں<br>16) Cement bricks سیمنٹ کی اینٹیں<br>17) Wood planks/shingles لکڑی کے تختے/شنگلز<br>18) Other, specify: دیگر، وضاحت کریں_________________
**Drinking Water Details پینے کے پانی کی تفصیلات**		
92.	What is the main source of drinking water for members of your household?<br><br>آپ کے گھر میں پینے کے پانی کا سب سے اہم ذریعہ کیا ہے ؟	1. Piped water into dwelling گھرمیں نلکا<br>2. Piped to yard/plot صحن میں نلکا<br>3. Handpump ہاتھ والا نلکا<br>4. Protected well محفوظ کنواں<br>5. Water from spring – Protected محفوظ چشمہ<br>6. Tube well or borehole ٹیوب ویل/ بورنگ کا پانی<br>7. Public tap/standpipe گلی /علاقہ کا نلکا<br>8. Tanker truck پانی کا ٹینکر<br>9. Cart with small tank ریڑھی کے ساتھ چھوٹی ٹنکی<br>10. Rainwater بارش کا پانی<br>11. Unprotected well غیر محفوظ کنواں<br>12. Water from spring – unprotected غیر محمفوظ چشمہ<br>13. Surface water (river/lake/pond/stream/Others) سطح والا پانی (دریا،جھیل ،ڈیم،تالاب/ ندی/نہر کا پانی)<br>14. Other, specify دیگر وضاحت کریں<br>________________
93.	House Ownership<br>آپ کے گھرکی مالکانہ حیثیت کیا ہے ؟	1) Rent کرایہ 2) Own اپنا 3) Heritage ورثہ
94.	Other Properties دیگر پراپرٹیز	a. House: گھر 1) Yes 2) No If Yes Number → [__] [__]<br>b. Shop: شاپ 1) Yes 2) No If Yes Number → [__] [__]<br>c. Other: دیگر1) Yes 2) No -------- If Yes specify____________<br>Number → [__] [__]
95.	Does any member of this household own any agricultural land?<br><br>کیا اس گھر میں رہنے والا کوئی فرد زرعی زمین کا مالک ہے؟	1) Yes, go to question 96<br>ہاں،تو سوال نمبر 96 پرجائیں<br>2) No, skip to question **97**<br>نہیں،تو سوال نمبر 97پرجائیں

96.	If yes, how many acres or canals of agricultural land do members of this household own?<br>اگر ہاں، تو اس گھر کے افراد کے پاس کتنے مربہ/ایکٹرپر مشتمل زمین ہے ؟	1) Acres ایکڑ ------------------------------ [__] [__] [__]<br>2) canals کنال ------------------------------ [__] [__] [__]<br>3) Don't know معلوم نہیں
**Information about Asset اثاثہ کے بارے میں معلومات**		
97.	Family Commute Details<br>گھر کے کسی فرد کے پاس یہ چیزیں موجود ہیں؟	1) None کوئی نہیں<br>2) Motorcycleموٹر سائیکل -------------------- [__] [__]<br>3) Bicycle سائیکل-------------------------------- [__] [__]<br>4) Animal drawn cart جانوروں سے چلائی جانے والی گاڑی -- [__] [__]<br>5) Car کار -- [__] [__]<br>6) Other(specify) دیگر وضاحت کریں____________[__] [__]
98.	Amenities سہولیات	1) Wi-Fi/Internet deviceوائی فائی/انٹرنیٹ ڈیوائس [__] [__]<br>2) Solar panel سولر پینل------------------------ [__] [__]<br>3) Electronic gadget الیکٹرانک گیجٹ -------- [__] [__]<br>4) UPS/Generator- UPS/جنریٹر------------- [__] [__]<br>5) Other دیگر ---------------------------------- [__] [__]
99.	Animal/Pet Details<br>جانوروں/پالتو جانوروں کی تفصیلات	1) Yes, go to question 100-ہاں، سوال نمبر 100 پر جائیں<br>2) No, go to next section 101 نہیں، اگلے سیکشن 101 پر جائیں-
100.	If yes, how many livestock, animals or poultry does your household own?<br>اگر ہاں تو آپ کے گھر میں کتنے مویشی جانور یا مرغیاں موجود ہیں؟<br>Ask for each listed animals, one by one and observation by enumerator Insert 000 if none<br>ایک ایک کر کے جانوروں کے نام پوچھیں اور شمار کنندہ مشاہدہ کرے<br>اگر کوئی نہیں تو 000 لکھیں	1) Cows, Bulls (مال مویشی (گائے اور بیل----- [__] [__]<br>2) Horses/Donkeys/ Mules گھوڑے/گدھے/خچر[__] [__]<br>3) Goats بکریاں ------------------------------------ [__] [__]<br>4) Sheepبھیڑ -- [__] [__]<br>5) Chickens /Poultry مرغیاں------------------ [__] [__]<br>6) Buffalo بھینسیں-------------------------------- [__] [__]<br>7) Camels اونٹ --------------------------------- [__] [__]
101.	Do you or any member of your household own any of the following items? [Use best estimate quantities]<br>کیا آپ یا آپ کے گھر کا کوئی فرد مندرجہ ذیل اشیاء میں سے کسی کا مالک ہے؟ [بہترین تخمینہ مقدار کا استعمال کریں]	1) Mobile phone موبائل فون------------------ [__] [__]<br>2) Refrigerator ریفریجریٹر-------------------- [__] [__]<br>3) Sofa/Chair صوفہ/کرسی -------------------- [__] [__]<br>4) Clock/Watch گھڑی/گھڑی------------------ [__] [__]<br>5) Iron لوہا ------------------------------------- [__] [__]<br>6) Washing Machine واشنگ مشین ----------- [__] [__]<br>7) Television ٹیلی ویژن ------------------------ [__] [__]<br>8) Computer کمپیوٹر-------------------------- [__] [__]<br>9) AC/Room Cooler AC/کمرے کا کولر------ [__] [__]<br>10) Water Boat/Ship پانی کی کشتی/جہاز------ [__] [__]<br>11) Radio ریڈیو-------------------------------- [__] [__]<br>12) Other, specify دیگر، وضاحت کریں_______[__] [__]
102.	**Household Monthly Income**<br>گھریلو ماہانہ آمدنی	|____|____|____|____|____|____|

Child Sample Details بچوں کے نمونے کی تفصیلات

103.	**Sample Type** نمونہ کی قسم	**Date Collected (DD/MM/YYYY)**<br>جمع کی تاریخ (سال- مہینہ – دن)	**Time Collected**<br>جمع کا وقت	**Barcode Scan**<br>بارکوڈ اسکن

104.	**Blood Sample** خون کا نمونہ	_ _/_ _/_ _ _ _	_ _ : _ _	
105.	**Stool Sample** پاخانے کا نمونہ	_ _/_ _/_ _ _ _	_ _ : _ _	
	**Mother Sample Details** والدہ کے نمونے کی تفصیلات			
106.	**Sample Type** نمونہ کی قسم	**Date Collected (DD/MM/YYYY)** جمع کی تاریخ (سال۔ مہینہ – دن)	**Time Collected** جمع کا وقت	**Barcode Scan** بارکوڈ اسکین
107.	**Blood Sample** خون کا نمونہ	_ _/_ _/_ _ _ _	_ _ : _ _	
108.	**Stool Sample** پاخانے کا نمونہ	_ _/_ _/_ _ _ _	_ _ : _ _	
109.	**Hair Sample** بالوں کا نمونہ	_ _/_ _/_ _ _ _	_ _ : _ _	
110.	**Breastmilk** چھاتی کا دودھ	_ _/_ _/_ _ _ _	_ _ : _ _	

	**Child MRI Details** بچوں کے ایم آر آئی کی تفصیلات		
		**Date of Scan (DD/MM/YYYY)** اسکین کی تاریخ (سال۔ مہینہ – دن)	**Time of Scan** اسکین کا وقت
111.	**MRI Scan** ایم آر آئی اسکین	_ _/_ _/_ _ _ _	_ _ : _ _

Form Completion فارم کی تکمیل

112. Provide any additional comments from the interview (optional) انٹرویو سے کوئی اضافی تبصرے فراہم کریں (اختیاری)

____
____
____
____

**113.CRF to be signed when complete by person who completed it** *Do not sign if any fields are empty* CRF مکمل ہونے پر دستخط کرنے والے شخص کے ذریعہ اسے مکمل کیا جائے۔ اگر کوئی فیلڈ خالی ہے تو دستخط نہ کریں۔	Name: نام __________________ ID/Code: آئی ڈی / کوڈ ____________ **Initials:**ابتدائیہ ________________	Date: تاریخ _ _/_ _/_ _ _ _ (DD / MM / YYYY) (سال۔ مہینہ – دن)	Time: وقت _ _ : _ _
