# Supplementary figures and images for: Association Between Human Milk-Targeted Metabolites and Maternal Characteristics: Targeted Metabolomic Profiling of Human Milk in Low-Income Settings
Source: Metabolites. 2026 Feb 28;16(3):162. doi: 10.3390/metabo16030162 (PMC13028068; doi:10.3390/metabo16030162)

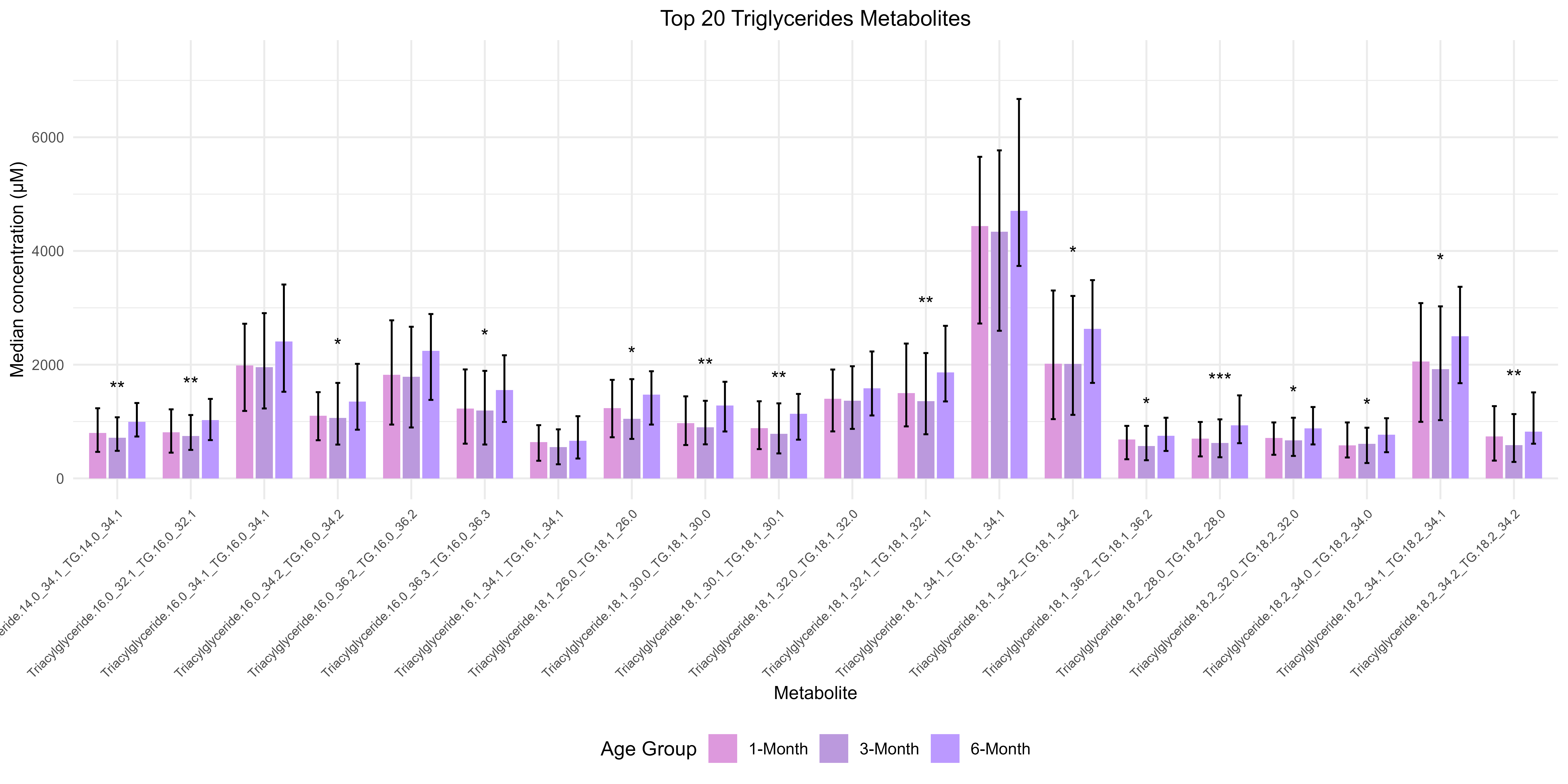

Supplement: Supplementary file 1 [file metabolites-16-00162-s001.zip › Figure S1.tif]

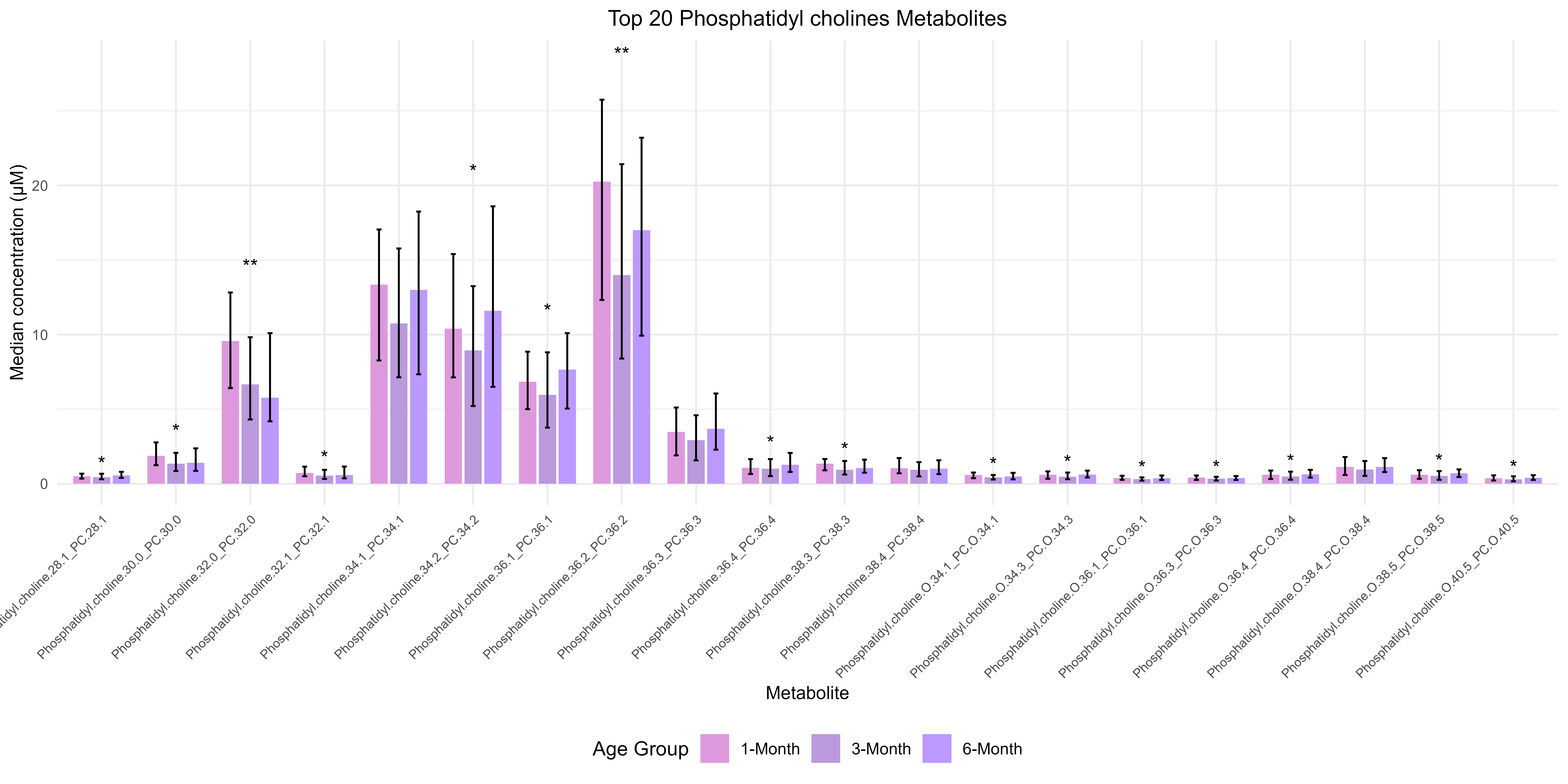

Supplement: Supplementary file 1 [file metabolites-16-00162-s001.zip › Figure S2.tif]
